# Cytokine-cytokine receptor interactions in the highly pathogenic avian influenza H5N1 virus-infected lungs of genetically disparate Ri chicken lines

**DOI:** 10.5713/ab.21.0163

**Published:** 2021-06-24

**Authors:** Thi Hao Vu, Yeojin Hong, Anh Duc Truong, Jiae Lee, Sooyeon Lee, Ki-Duk Song, Jihye Cha, Hoang Vu Dang, Ha Thi Thanh Tran, Hyun S. Lillehoj, Yeong Ho Hong

**Affiliations:** 1 Department of Animal Science and Technology, Chung-Ang University, Anseong 17546, Korea; 2 Department of Biochemistry and Immunology, National Institute of Veterinary Research, Hanoi 100000, Vietnam; 3 Department of Animal Biotechnology, College of Agricultural and Life Sciences, Jeonbuk National University, Jeonju, 54896, Korea; 4 Animal Genomics and Bioinformatics Division, National Institute of Animal Science, RDA, Wanju 55365, Korea; 5 Animal Biosciences and Biotechnology Laboratory, Agricultural Research Services, United States Department of Agriculture, Beltsville, MD 20705, USA

**Keywords:** Cytokine-cytokine Receptor, H5N1, Highly Pathogenic Avian Influenza Virus (HPAIV), Ri Chickens, RNA Sequencing

## Abstract

**Objective:**

The highly pathogenic avian influenza virus (HPAIV) is a threat to the poultry industry as well as the economy and remains a potential source of pandemic infection in humans. Antiviral genes are considered a potential factor for HPAIV resistance. Therefore, in this study, we investigated gene expression related to cytokine-cytokine receptor interactions by comparing resistant and susceptible Ri chicken lines for avian influenza virus infection.

**Methods:**

Ri chickens of resistant (*Mx*/A; *BF2*/B21) and susceptible (*Mx*/G; *BF2*/B13) lines were selected by genotyping the Mx dynamin like GTPase (*Mx*) and major histocompatibility complex class I antigen BF2 genes. These chickens were then infected with influenza A virus subtype H5N1, and their lung tissues were collected for RNA sequencing.

**Results:**

In total, 972 differentially expressed genes (DEGs) were observed between resistant and susceptible Ri chickens, according to the gene ontology and Kyoto encyclopedia of genes and genomes pathways. In particular, DEGs associated with cytokine-cytokine receptor interactions were most abundant. The expression levels of cytokines (interleukin-1β [IL-1β], IL-6, IL-8, and IL-18), chemokines (C-C Motif chemokine ligand 4 [CCL4] and CCL17), interferons (IFN-γ), and IFN-stimulated genes (*Mx1*, *CCL19*, 2′-5′-oligoadenylate synthase-like, and protein kinase R) were higher in H5N1-resistant chickens than in H5N1-susceptible chickens.

**Conclusion:**

Resistant chickens show stronger immune responses and antiviral activity (cytokines, chemokines, and IFN-stimulated genes) than those of susceptible chickens against HPAIV infection.

## INTRODUCTION

Avian influenza (AI) is a virus of the genus influenzavirus A in the *Orthomyxoviridae* family of viruses [1]. Based on genetics and disease severity, AI viruses are classified either as highly pathogenic avian influenza (HPAI) or lowly pathogenic AI [2]. In particular, a high number of deaths were observed in birds caused by HPAI [3]. Moreover, the influenza A virus subtype H5N1, type of HPAI virus (HPAIV), is a threat to the poultry industry and the economy and remains a potential source of pandemic infection in humans [4].

Mx dynamin like GTPase (Mx) proteins are members of the dynamin family of large GTPases. They prevent viral RNA replication by inhibiting the trafficking or activity of viral polymerases [5]. Several previous reports showed that only the asparagine (Asn-AAT) polymorphism at the 631st position triggered antiviral activity, while Mx proteins carrying a serine (Ser-AGT) at that position did not suppress viral growth [6]. In addition, the major histocompatibility complex (MHC) haplotype can affect the antiviral activity of the host [7]. Previous research has shown a significant association of the major histocompatibility complex class I antigen BF2 (BF2) haplotype in chicken MHC class I with resistance or susceptibility to a number of pathogens, including Marek virus [8], and AI virus [9]. Furthermore, chickens with the *BF2*-B13 haplotype had higher mortality than those that have the *BF2*-B21 haplotype [9].

One of the key determinants of the severity and outcome of AI virus infection is the regulation of the host innate immune response [10]. Cytokines and chemokines play crucial roles in the balance of the immune system. Previous studies in various animals have shown that tissue damage and host death are caused by cytokine and chemokine dysregulation [11]. In chickens, HPAIV H5N1 induced the excessive expression of cytokines and chemokines in lung tissues [12]. Furthermore, cytokine-cytokine receptor interactions were significantly increased in Fayoumi and Leghorn chicken lines after infection with HPAIV [13]. The regulation of cytokines and chemokines has been shown to be important in host defense against HPAIV.

In this study, we used Ri chickens, a local chicken breed in Vietnam, as an experimental animal [14]. Chickens resistant or susceptible to HPAIV were distinguished by genotyping their *Mx* and *BF2* genes. We infected Ri chickens with HPAIV H5N1 and analyzed gene expression patterns in the lung tissue using high-throughput RNA sequencing. In particular, we analyzed the expression of genes related to cytokine-cytokine receptor interactions between resistant and susceptible Ri chickens.

## MATERIALS AND METHODS

### Experimental animals and HPAIV infection

Fourteen-day-old post-hatching Ri chickens were kept in specific-pathogen-free conditions and observed daily for signs of disease and mortality. The care and experimental use of the chickens were approved by the Ministry of Agriculture and Rural Development of Vietnam (TCVN 8402: 2010/TCVN 8400-26:2014).

For the *Mx* gene, Ri chickens that have the non-synonymous adenine single nucleotide polymorphism (SNP) at residue 631 were genotyped as resistant while guanine was genotyped as susceptible (Supplementary Figure S1). In addition, for the *BF2* gene, those that had the B21 genotype were determined to be resistant and those that had the B13 genotyped were susceptible. Taking these together, HPAIV-resistant Ri chickens had the genotype *Mx*(A)/*BF2* (B21) and HPAIV-susceptible Ri chickens had the genotype *Mx*(G)/*BF2*(B13). For HPAIV infection, a total of 10 Ri chickens (five resistant and five susceptible) were inoculated intranasally with the collected allantoic fluid including 10^4^ egg infectious dose (EID50) of A/duck/Vietnam/QB1207/2012 (H5N1) based on the OIE guidelines [15]. All experiments, including chicken management, HPAIV infection, and sample collection, were conducted at the National Institute of Veterinary Research, Vietnam.

### Sample collection and total RNA extraction

At three days post-infection, the lung tissues were collected from five chickens per group, according to the WHO Manual on Animal Influenza Diagnosis and Surveillance. Total RNA from lung tissue was extracted using the TRIzol reagent (Invitrogen, Carlsbad, CA, USA) according to the manufacturer’s instructions.

### High-throughput RNA sequencing and data analysis

Potentially existing sequencing adapters and low-quality bases in the raw reads were trimmed using Skewer version 0.2.2. The cleaned high-quality reads after trimming the low-quality bases and sequencing adapters were mapped to the reference genome using STAR software version 2.5. Since the sequencing libraries were prepared in a strand-specific manner using the Illumina strand-specific library preparation kit (Illumina Way, San Diego, CA, USA), the strand-specific library option was applied in the mapping process. The gene annotation of the chicken reference genome gg6 from the UCSC genome (https://genome.ucsc.edu) and the corresponding expression values were calculated as fragments per kilobase of transcript per million fragments mapped (FPKM). The differentially expressed genes (DEGs) between the two selected biological conditions were analyzed using the Cuffdiff software in the Cufflinks package version 2.2.1. Assignment to significance was determined using a threshold. The default threshold was set as |fold-change| ≥2 and p-value≤0.05. To gain insight into the biological roles of the genes differentially expressed between the susceptible and resistant lines, a gene set overlapping test was performed between the analyzed DEGs and functionally categorized genes, including the biological processes determined from the gene ontology (GO), Kyoto encyclopedia of genes and genomes (KEGG) pathways, and other functional gene sets using g: Profiler version 0.6.7.

### Cytokine-cytokine receptor interaction enrichment analysis

KEGG pathway analysis of the DEGs revealed that most DEGs were related to cytokine-cytokine receptor interactions. Therefore, cytokine-cytokine receptor interactions were analyzed in this study. We used the Search and Color Pathway tool of KEGG Mapper (https://www.genome.jp/kegg/tool/map_pathway2.html) to identify the upregulated or downregulated genes between resistant and susceptible Ri chicken lines. The mutual interactions of the cytokine signaling pathway-related genes were analyzed using the Pathway Studio program (Ariadne Genomics, Rockville, MD, USA).

### Quantitative real-time polymerase chain reaction validation for cytokine-cytokine receptor interaction genes

Quantitative real-time polymerase chain reaction (qRT-PCR) was performed to verify the DEGs obtained from RNA sequencing. Before cDNA synthesis, 2 μg of total RNA (R1D3, R2D3, R3D3, and R5D3 for resistant line and S1D3, S3D3, and S5D3 for susceptible line) was treated with 1.0 U DNase I (Thermo Fisher Scientific, Waltham, MA, USA) to remove potentially contaminating genomic DNA. cDNA synthesis was performed using a RevertAid First Strand cDNA Synthesis Kit (Thermo Fisher Scientific, USA) according to the manufacturer’s instructions. The primer sequences of 15 genes and the housekeeping gene glyceraldehyde-3-phosphate dehydrogenase (GAPDH) were designed using Primer-BLAST (http://www.ncbi.nlm.nih.gov/tools/primer-blast/) (Table 1). The qRT-PCR was performed in a LightCycler 96 system (Roche, Indianapolis, IN, USA) using the AMPI-GENE qPCR Green Mix Hi-Rox kit (Enzo Life Sciences, Inc., Farmingdale, NY, USA) according to the manufacturer’s recommendations. Relative gene expression was calculated using the 2^−ΔΔCt^ method after normalization with chicken GAPDH [16]. All experiments were performed independently in triplicates.

### Statistical analysis

Statistical analysis was carried out using SPSS software (IBM, SPSS 26.0 for Windows, Chicago, IL, USA), and p<0.05 was considered as a statistically significant expression level using Student’s t-test. All qRT-PCR experiments were replicated independently three times, and the mean±standard error of the mean values for each group were reported.

## RESULTS

### Analysis of RNA sequencing

After H5N1 infection, we observed that chickens had ruffled hair; pulmonary edema, pneumorrhagia, and congestive lung. Among the lung tissue samples obtained from resistant and susceptible Ri chickens three days post H5N1 infection, six samples passed the quality control check and were subject high-throughput RNA sequencing (Table 2).

Table 2 shows the statistics of the raw and clean reads of individual sample transcriptomes after sequence processing and analysis. The six libraries produced approximately 6 GB worth of cDNA sequences per chicken. After data filtering, approximately 21 million clean reads were obtained for each sample from the resistant and susceptible lines.

The number of mapped reads, percentages, and transcripts is shown in Table 3. More than 88.5% of the filtered reads from each library were mapped to the reference genome.

### Gene ontology functional enrichment and KEGG pathway analysis

**Figure 1:**
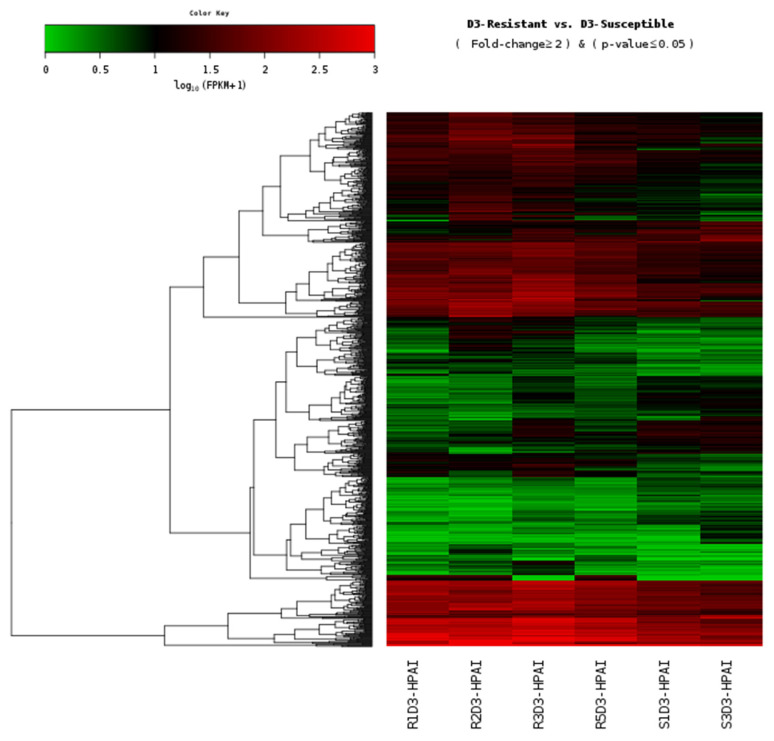
Heat map of the individual H5N1-infected samples from the lung tissue of resistant and susceptible Ri chickens at three days post-infection. A green color indicates DEGs that have higher expression levels in resistant chickens while a red color indicates DEGs that have lower expression levels in resistant chickens, as calculated from the expression values in log10 (FPKM) units. H5N1, influenza A virus subtype H5N1; DEGs, differentially expressed genes; FPKM, fragments per kilobase of exon model per million reads mapped.

The calculated FPKM values were used to compare the mRNA expression levels between resistant and susceptible Ri chickens (Figure 1). Among the 972 DEGs, 309 genes (32%) were significantly upregulated and 663 genes (68%) were downregulated in susceptible chickens compared to the resistant chicken lines (Supplementary Table S1).

**Figure 2:**
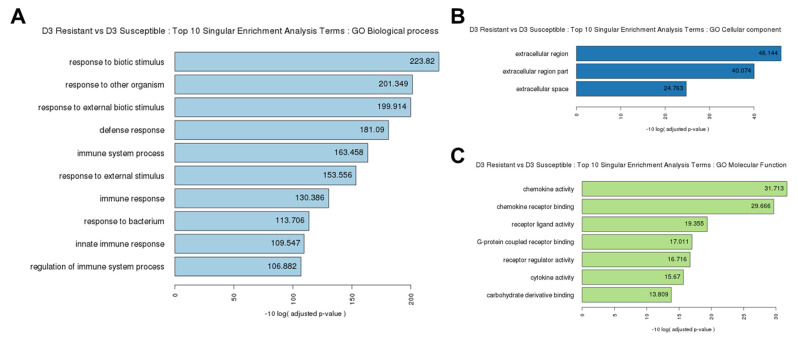
Gene ontology (GO) functional analysis. The enriched biological terms include: (A) Top 10 SEA: GO Biological process, (B) Top 10 SEA: GO Cellular component, and (C) Top 10 SEA: GO Molecular function from the 972 DEG in Ri chicken lines obtained using the criteria (|fold-change| ≥2) ∩ (p≤0.05). GO, gene ontology; SEA, singular enrichment analysis terms; DEG, differentially expressed genes.

**Figure 3:**
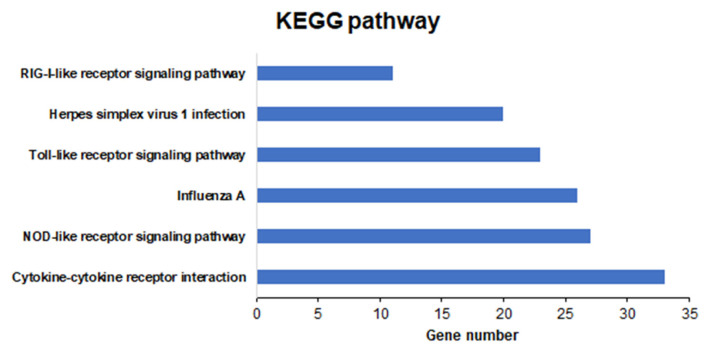
Six Kyoto encyclopedia of genes and genomes functional pathways of differentially expressed genes in Ri chickens of the selected differentially expressed genes.

To explore the function of these DEGs, we performed GO and KEGG pathway analyses. Among the 972 DEGs, 114 were associated with biological processes, seven were associated with molecular functions, and three were associated with cellular components (Figure 2; Supplementary Table S2). KEGG pathway analysis revealed six categories of immune-related pathways, including cytokine-cytokine receptor interactions (33 DEGs), nucleotide-binding oligomerization domain like receptor (NOD-like receptor) signaling pathway (27 DEGs), influenza A (26 DEGs), Toll-like receptor signaling pathway (23 DEGs), herpes simplex infection (20 DEGs), and RIG-I-like receptor signaling pathway (11 DEGs) (Figure 3). Most of the DEGs were related to cytokine-cytokine receptor interactions.

### Analysis of cytokine-cytokine receptor interactions related to the DEGs

**Figure 4:**
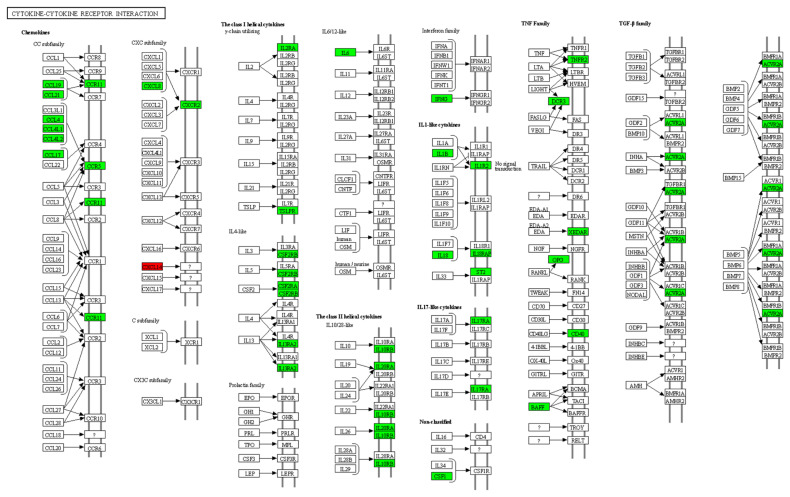
Location of differentially expressed genes associated with cytokine-cytokine receptor interactions in the lungs of resistant H5N1-infected Ri chickens compared with susceptible chickens. The location of 33 significantly different expressed genes were marked. A green color indicates DEGs that have higher expression levels in resistant chickens while a red color indicates DEGs that have lower expression levels in resistant chickens (CXCL14). H5N1, influenza A virus subtype H5N1; DEG, differentially expressed genes.

Based on the RNA sequencing results and subsequent mapping to the KEGG pathway database, 33 DEGs were involved in cytokine-cytokine receptor interactions (Supplementary Table S3). Among them, 32 genes were significantly downregulated, and one gene was significantly upregulated in susceptible chickens compared to the resistant chicken lines (Figure 4).

**Figure 5:**
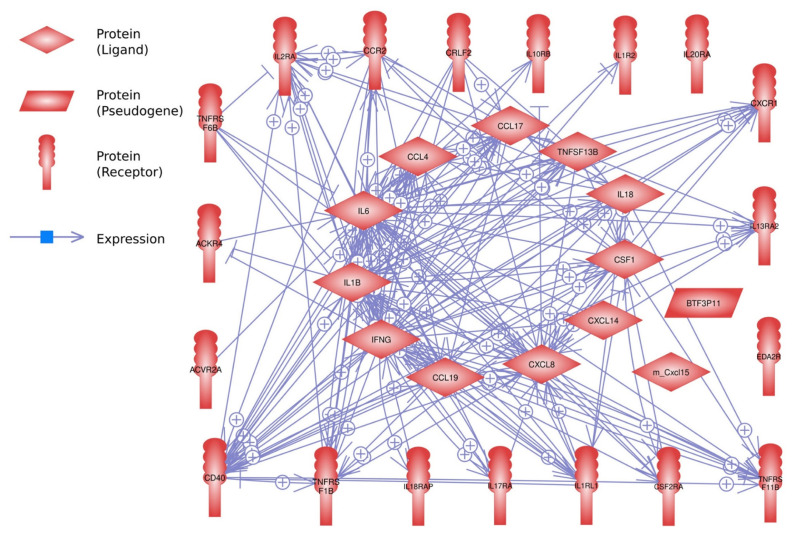
Interactions of the 33 differentially expressed genes in the lungs of Ri chicken lines. This interaction analysis was conducted using the Pathway Studio program.

For the interaction analysis, 32 DEGs out of 33 genes were identified using the Pathway Studio program (Figure 5). Of these, 28 genes showed strong interactions with each other, but four genes showed no interaction.

### Quantitative real-time polymerase chain reaction analysis of genes associated with cytokine-cytokine receptor interactions

**Figure 6:**
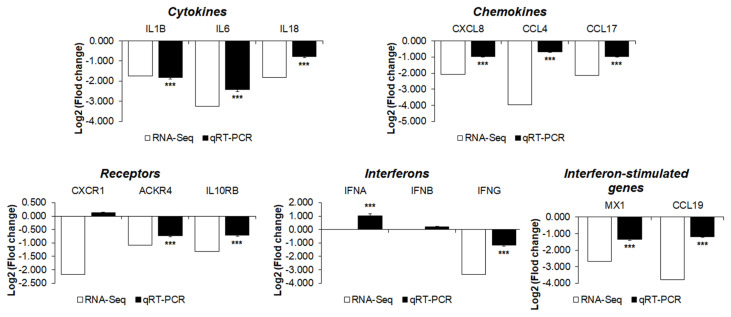
Analysis of the expression of genes associated with cytokine-cytokine receptor interactions in resistant H5N1-infected chickens compared with susceptible H5N1-infected chickens. The expression patterns of these genes were compared between real-time qPCR and RNA sequencing data. Relative quantitation data of qRT-PCR are represented as mean±SEM normalized to GAPDH using the 2^−ΔΔCt^ method. H5N1, influenza A virus subtype H5N1; qRT-PCR, quantitative real-time polymerase chain reaction; SEM, standard error of the mean; GAPDH, glyceraldehyde-3-phosphate dehydrogenase. Data are expressed as mean±SEM of three independent experiments: * p<0.05, ** p<0.01, and *** p<0.001.

To validate the RNA sequencing results, the expression levels of 14 cytokines, chemokines, and receptors of the 33 DEGs between the two chicken lines were quantitatively determined via qRT-PCR (Figure 6). After H5N1 infection, the expression levels of interleukin-1β (IL-1β), IL-6, IL-18, C-C Motif chemokine ligand 4 (CCL4), CCL17, interleukin 8 (CXCL8, IL-8), interleukin 10 receptor subunit beta (IL10RB), atypical chemokine receptor 4 (ACKR4), interferon-γ (IFN-γ), CCL19, and Mx1 were significantly lower in susceptible Ri chickens than in resistant chickens. These results indicate that the expression levels of these genes obtained by qRT-PCR are consistent with RNA-seq results.

## DISCUSSION

In this study, we analyzed the transcriptome profiles of Ri chickens infected with HPAIV H5N1 using RNA sequencing. HPAIV-resistant and HPAIV-susceptible Ri chicken lines were selected based on their *Mx* and *BF2* genotypes and were infected with HPAIV H5N1. RNA sequencing was conducted after infection and 972 DEGs were identified after comparing the transcriptome profiles of lung tissue obtained between resistant and susceptible chickens. KEGG analysis revealed that most of the DEGs were related to cytokine-cytokine receptor interactions.

Avian influenza viral pathogen-associated molecular patterns are recognized by host pattern recognition receptors (PRRs). Viral double-stranded RNA (dsRNA) and cytosine-guanosine oligodeoxynucleotides, which form during the replication of AIV, are recognized by toll-like receptor 3 (TLR3) [17] and TLR21 [18], respectively, through adaptor proteins such as TIR-domain-containing adapter-inducing interferon and myeloid differentiation primary response 88 (MyD88), respectively [19]. These adaptors activate the transcription factor interferon regulatory factor 7 (IRF7) and nuclear factor kappa B (NF-κB) to induce cytokines, chemokines, and IFN-stimulated genes [20]. High expression levels of PRRs, MyD88, IRF7, and NF-κB were previously observed in H5N1-infected chickens [12]. Our results showed that the expression levels of TLR3, TLR21, IRF7, and MyD88 were higher in resistant chickens compared to susceptible chickens (Supplementary Table S1); therefore, we suggest that resistant Ri chickens respond more sensitively to HPAIV H5N1 infection than susceptible Ri chicken lines through increased expression of TLR3, TLR21, IRF7, and MyD88.

Pro-inflammatory cytokines cause inflammation and recruit other immune cells to the site of infection [21]. High expression levels of pro-inflammatory cytokines and chemokines have been found in humans and duck infected with H5N1 [10,11]. Similarly, high expression levels of pro-inflammatory cytokines and chemokines, including IL-1β, IL-6, IL-8, IL-18, CCL4, CCL17, and IFN-γ, were reported in chickens infected with H5N1 [12]. A significant increase in the levels of cytokines and chemokines exacerbates the inflammatory response, leading to apoptosis, multi-organ failure, and host death [22]. However, HPAIV infection was lethal in mice lacking tumor necrosis factor and IL-1 receptors and inhibition of the cytokine response does not protect mice against H5N1 influenza infection [23]. In this study, expression levels of pro-inflammatory cytokines and chemokines such as IL-1β, IL-6, IL-8, IL-18, CCL4, CCL17, IFN-γ, and receptors were upregulated in resistant chickens, compared to susceptible chickens, after HPAIV H5N1 infection (Figure 6). However, the induction of cytokines and chemokines did not cause Ri chickens to die during experimentation. Therefore, we suggest that cytokine and chemokine induction is necessary to protect the host against HPAIV infection.

Type I interferons (IFN-α and IFN-β) trigger the expression of IFN-stimulated genes. IFNs and IFN-stimulated genes can inhibit viral replication by blocking virus entry into the host cells, binding to viral RNA to stop translation, and regulating host antiviral responses [24]. IFN-γ directly or indirectly inhibits viral replication by strictly regulating the production of nitric oxide or interfering with the onset of the RNase L pathway [25]. Moreover, several studies have shown that IFN-stimulated genes have antiviral activity [26, 27]. The *Mx* gene inhibits the trafficking and activity of viral polymerases [5]. Viperin (RSAD2) inhibits newly synthesized influenza virions [28]. The CCL19, CCL21, and CCR7 chemokine axes are homeostatic [29]. Chicken interferon-inducible 2′-5′-oligoadenylate synthase-like (OASL) and RNase L restrict both viral and cellular RNA, preventing viral genome replication [30]. Wild-type duck OASL inhibits the replication of a variety of RNA viruses *in vitro*, including the influenza virus [25]. Protein kinase R (EIF2AK2) inhibits the translation of viral mRNAs, including those from influenza A viruses [26]. Interferon-induced proteins of the tetratricopeptide repeats (IFIT) protein family sequester viral nucleic acids [31]. Furthermore, the clinical results were ameliorated in chIFIT5-transgenic chickens after treatment with HPAIV and Newcastle disease virus [27]. As our results showed, although IFN-α and IFN-β were not found in the DEGs, we observed a higher expression of IFN-γ and IFN-stimulated genes (*Mx*, *CCL19*, *OASL*, *RSAD2*, *EIF2AK2*, and *IFIT5*) in the resistant Ri chickens compared to the susceptible ones (Supplementary Table S1). Therefore, we suggest that the antiviral response of resistant chickens is higher than that of susceptible chickens.

In summary, by using RNA sequencing and qRT-PCR, we evaluated the differential expression of genes associated with cytokine-cytokine receptor interactions from the lung tissue of resistant and susceptible H5N1-infected Ri chickens. Interestingly, the expression of PRRs, cytokines, chemokines, and IFN-stimulated genes in resistant chickens were higher than those in susceptible chickens. These results suggest that resistant Ri chickens show a higher antiviral activity compared to susceptible Ri chickens, and this antiviral activity may be related to the expression of antiviral genes. Therefore, we propose that more studies on Ri chickens be done to compare their resistance and susceptibility to HPAIV infections. With this, there will be more studies to support advancing the development of chicken lines with disease resistance genes.

## Figures and Tables

**Table 1:** Primer sequences used for quantitative real-time polymerase chain reaction

Genes		Primer sequence	Product (bp)	GenBank accession No.
*GAPDH*	F	TGC TGC CCA GAA CAT CAT CC	142	NM_204305
	R	ACG GCA GGT CAG GTC AAC AA		
*IL-1β*	F	TGC CTG CAG AAG AAG CCT CG	137	NM_204524.1
	R	CTC CGC AGC AGT TTG GTC AT		
*IL6*	F	GCA GGA CGA GAT GTG CAA GA	131	NM_204628.1
	R	ATT TCT CCT CGT CGA AGC CG		
*IL8*	F	GGC TTG CTA GGG GAA ATG A	200	NM_205498.1
	R	AGC TGA CTC TGA CTA GGA AAC TGT		
*IL18*	F	GGA ATG CGA TGC CTT TTG	264	NM_204608.1
	R	ATT TTC CCA TGC TCT TTC TCA		
*CCL4*	F	CTT CAC CTA CAT CTC CCG GC	145	NM_001030360.2
	R	CTG TAC CCA GTC GTT CTC GG		
*CCL17*	F	TCT CGA AGC GCT GAA GAG TT	118	NM_204596.1
	R	TTT CAC CCA AGG TGC GTT TG		
*CCL19*	F	TGC CTT AGT CTC CTG GTG CT	177	NM_001302168.1
	R	CTT TGC AGT GAT GAA CAC GGT		
*IFN-α*	F	GAG CAA TGC TTG GAC AGC AG	183	GU119896.1
	R	GAG GTT GTG GAT GTG CAG GA		
*IFN-β*	F	CTT GCC CAC AAC AAG ACG TG	139	NM_001024836.1
	R	TGT TTT GGA GTG TGT GGG CT		
*IFN-γ*	F	AAC AAC CTT CCT GAT GGC GT	106	NM_205149.1
	R	TGA AGA GTT CAT TCG CGG CT		
*MX1*	F	AGC CAT AGA ACA AGC CAG AA	127	NM_204609.1
	R	GGT ACT GGT AAG GAA GGT GG		
*CXCR1*	F	TTA CGC TGA CGA ACT CTT GG	109	NM_001282432.1
	R	TTC ATT ACG GCA TGG GGA AG		
*IL10RB*	F	CCA CCC ATA ACG GTG TAA CT	132	XM_015299252.2
	R	AAA CAT GCT TGG TGA ATC GG		
*ACKR4*	F	CAT CAT GCA CCT AGC CAT TG	108	XM_025147497.1
	R	GTG AGC TTG CAC ATT GAG TT		

*GAPDH*, glyceraldehyde-3-phosphate dehydrogenase; *IL-1β*, interleukin-1β; *CCL4*, C-C Motif chemokine ligand 4; *IFN-α*, interferons; *MX1*, MX dynamin like GTPase 1; *CXCR1*, C-X-C motif chemokine receptor 1; *IL10RB*, interleukin 10 receptor subunit beta; *ACKR4*, atypical chemokine receptor 4.

**Table 2:** Summary of raw reads and clean reads of lung tissue samples obtained from highly pathogenic avian influenza virus-infected chickens from dpi 3 datasets

Sample name	Sample group	Raw reads				Clean reads			
	
		# Read pairs	Yield (bp)	*% ≥ Q30 Bases	Mean	# Read pairs	Yield (bp)	*% ≥ Q30 Bases	Mean
R1D3	Resistant	19,744,467	5,923,340,100	88.5	Q34.6	19,744,385	5,886,928,036	88.7	Q34.7
R2D3	Resistant	22,316,549	6,694,964,700	88.9	Q34.7	22,316,413	6,629,979,998	89.3	Q34.8
R3D3	Resistant	21,391,077	6,417,323,100	89	Q34.7	21,390,906	6,353,660,222	89.3	Q34.8
R5D3	Resistant	22,038,235	6,611,470,500	90.2	Q34.9	22,038,054	6,438,285,162	90.9	Q35.1
S1D3	Susceptible	21,399,608	6,419,882,400	90.7	Q35.1	21,399,449	6,350,528,440	91	Q35.1
S3D3	Susceptible	22,097,437	6,629,231,100	90.4	Q35.0	22,097,302	6,582,960,388	90.6	Q35.1

**Table 3:** Sequence alignment of the highly pathogenic avian influenza virus-infected samples

Sample name	Sample group	Mapping rate (%)	Unique mapped	Multiple mapped	Total unmapped	Unmapped by too short alignment (%)
R1D3	Resistant	91.37	17,247,800	793,620	1,702,965	8.23
R2D3	Resistant	88.53	18,571,703	1,186,083	2,558,627	11.13
R3D3	Resistant	90.53	18,311,414	1,053,231	2,026,261	9.1
R5D3	Resistant	89.13	18,672,789	969,647	2,395,618	10.39
S1D3	Susceptible	92.58	19,035,119	775,561	1,588,769	7.1
S3D3	Susceptible	92.66	19,810,851	664,829	1,621,622	6.99

## SUPPLEMENTARY MATERIAL

**Supplementary Table S1.** List of 972 differentially expressed genes selected using the criteria (|fold-change| ≥2) and (p≤0.05) from D3-Resistant and D3-Susceptible chickens **Supplementary Table S2.** Gene ontology enrichment of sequence data obtained from the lung tissue of H5N1-resistant and -susceptible chickens, with p<0.05 and Log2-transformation of the normalization of resistant/susceptible ≥2 **Supplementary Table S3.** List of all genes related to the cytokine-cytokine receptor interaction pathways obtained from the lung tissue of resistant and susceptible H5N1-infected Ri chicken lines (p-value). These data show the changes in gene expression of 190 genes after H5N1 infection **Supplementary Figure S1.** Sequencing analysis of the *Mx* gene in resistant and susceptible Ri chickens
