# Supplementary material for: Cytokine-cytokine receptor interactions in the highly pathogenic avian influenza H5N1 virus-infected lungs of genetically disparate Ri chicken lines
Source: Anim Biosci. 2021 Jun 24;35(3):367–76. doi: 10.5713/ab.21.0163 (PMC8902228; doi:10.5713/ab.21.0163)
Supplement: Supplementary file 1 [file ab-21-0163-suppl.pdf]

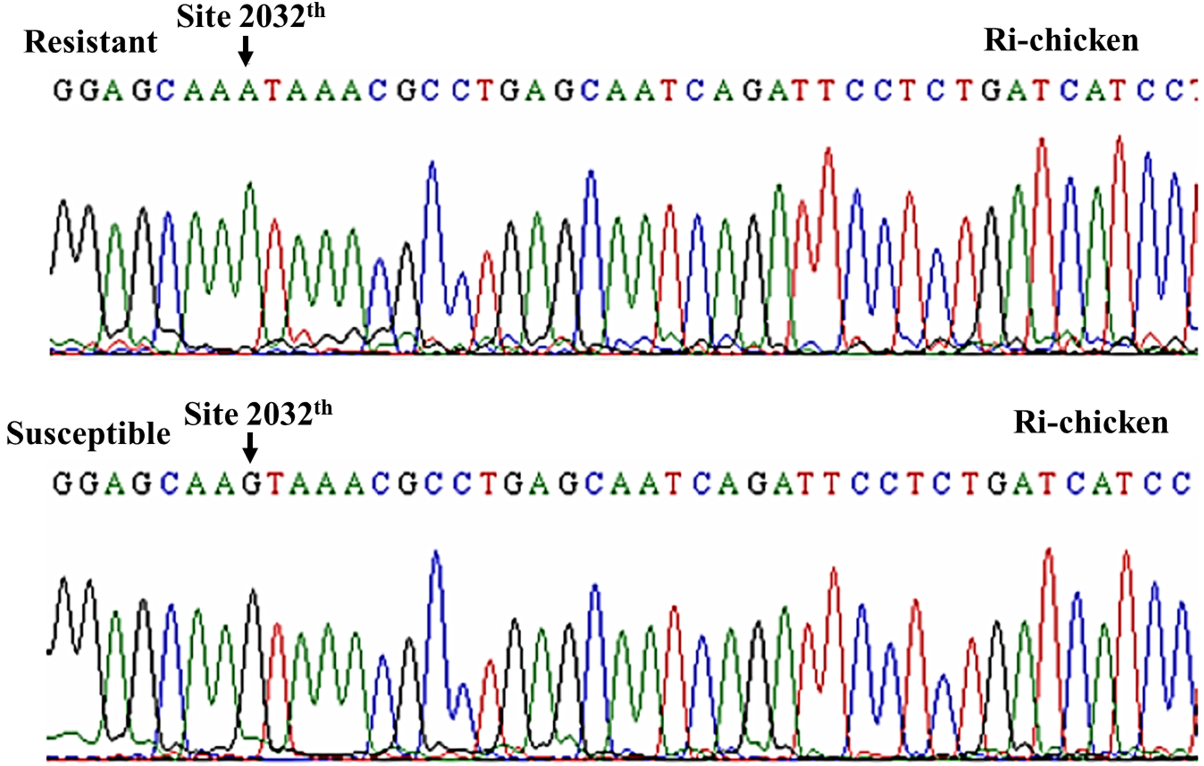
Resistant
Site 2032th
Ri-chicken
GGAGCAAATAAACGCCTGAGCAATCAGATTCCTCTGATCATCC
Susceptible
Site 2032th
Ri-chicken
GGAGCAAGTAAACGCCTGAGCAATCAGATTCCTCTGATCATCC

Table S1. 972 differentially expressed genes which have been selected by (|fold-change| ≥ 2) and (p-value ≤ 0.05) from D3-Resistant vs. D3-Susceptible

Gene Symbol	Full descriptions of the gene	D3-Resistant	D3-Susceptible	Log2(FC)	P-value
HINTW	histidine triad nucleotide binding protein W [Source:NCBI gene;Acc:395423]	368.806	0.410063	-9.8128	0.00535
ENSGALG00000039023	Nipped-B homolog-like [Source:NCBI gene;Acc:427025]	15.1276	0.069325	-7.76959	0.00025
ENSGALG00000033705	transitional endoplasmic reticulum ATPase-like [Source:NCBI gene;Acc:430766]	26.3674	0.133898	-7.62148	0.0006
ENSGALG00000030382	Golgi phosphoprotein 3-like [Source:NCBI gene;Acc:107049174]	16.5626	0.151352	-6.77388	0.03335
ENSGALG00000053121	chemokine ah221 [Source:NCBI gene;Acc:417536]	82.7435	2.05667	-5.33026	0.00015
ENSGALG00000031355	E3 ubiquitin-protein ligase NEDD4-like [Source:NCBI gene;Acc:107055431]	2.47342	0.07866	-4.97473	0.03525
ENSGALG00000053052	immunoglobulin superfamily member 1-like [Source:NCBI gene;Acc:112531168]	1.834	0.094161	-4.28372	0.0325
FKBP5	FK506 binding protein 5 [Source:NCBI gene;Acc:421186]	152.328	9.38036	-4.02139	0.00005
OVCH2	ovochymase 2 (gene/pseudogene) [Source:HGNC Symbol;Acc:HGNC:29970]	3.4278	0.213506	-4.00493	0.0061
MLKL	mixed lineage kinase domain like pseudokinase [Source:HGNC Symbol;Acc:HGNC:26617]	8.52434	0.531538	-4.00334	0.00065
CCL4	C-C motif chemokine ligand 4 [Source:NCBI gene;Acc:395468]	55.9887	3.57791	-3.96795	0.00005
RSAD2	radical S-adenosyl methionine domain containing 2 [Source:NCBI gene;Acc:428650]	1651.16	108.44	-3.92851	0.00005
CCL19	C-C motif chemokine ligand 19 [Source:NCBI gene;Acc:427406]	123.19	8.98977	-3.77646	0.00005
ENSGALG00000013033	carboxymethylenebutenolidase homolog [Source:NCBI gene;Acc:420928]	15.4164	1.20721	-3.67472	0.0007
PATL2	PAT1 homolog 2 [Source:HGNC Symbol;Acc:HGNC:33630]	16.9727	1.43544	-3.56365	0.039
HNF4beta	hepatic nuclear factor 4beta [Source:NCBI gene;Acc:415820]	1.65732	0.141369	-3.55131	0.0358

IFIT5	interferon induced protein with tetratricopeptide repeats 5 [Source:NCBI gene;Acc:423790]	7000.15	605.404	-3.53142	0.00835
S100A12	S100 calcium binding protein A12 [Source:NCBI gene;Acc:426356]	1011.11	88.8251	-3.50883	0.0353
CRH	corticotropin releasing hormone [Source:NCBI gene;Acc:404297]	3.92882	0.34576	-3.50625	0.04675
SOCS1	suppressor of cytokine signaling 1 [Source:NCBI gene;Acc:416630]	171.705	15.6146	-3.45896	0.00005
ENSGALG00000045115	Fc fragment of IgE receptor II [Source:NCBI gene;Acc:100858751]	69.7543	6.8575	-3.34653	0.03125
IFNG	interferon gamma [Source:NCBI gene;Acc:396054]	7.20664	0.713043	-3.33727	0.0031
PDK4	pyruvate dehydrogenase kinase 4 [Source:NCBI gene;Acc:420570]	94.5559	9.37111	-3.33488	0.00005
TRANK1	tetratricopeptide repeat and ankyrin repeat containing 1 [Source:HGNC Symbol;Acc:HGNC:29011]	944.103	95.4998	-3.30537	0.00005
CHIR-B6	Gallus gallus immunoglobulin-like receptor CHIR-B6 (CHIR-B6), mRNA. [Source:RefSeq mRNA;Acc:NM_001318406]	3.23139	0.334558	-3.27183	0.04995
IL6	interleukin 6 [Source:NCBI gene;Acc:395337]	5.21079	0.544259	-3.25914	0.0026
SERPINB10B	serpin peptidase inhibitor, clade B (ovalbumin), member 10 B [Source:NCBI gene;Acc:395715]	282.673	30.3252	-3.22055	0.00005
OLFML1	olfactomedin like 1 [Source:HGNC Symbol;Acc:HGNC:24473]	112.044	12.0772	-3.21371	0.00005
UPK3A	uroplakin 3A [Source:HGNC Symbol;Acc:HGNC:12580]	1.74666	0.189664	-3.20308	0.03855
RASD1	ras related dexamethasone induced 1 [Source:NCBI gene;Acc:416507]	11.9954	1.32226	-3.1814	0.00025
ENSGALG00000051203	MAS-related GPR, member H [Source:NCBI gene;Acc:425998]	10.2518	1.1397	-3.16915	0.00015
ENSGALG00000053860	mycocerosic acid synthase-like [Source:NCBI gene;Acc:420486]	2.27129	0.260301	-3.12526	0.00325
LHFPL5	lipoma HMGIC fusion partner-like 5 [Source:NCBI gene;Acc:378916]	13.4687	1.54856	-3.12061	0.0002
KCNJ5	potassium voltage-gated channel subfamily J member 5 [Source:HGNC Symbol;Acc:HGNC:6266]	32.6313	3.86203	-3.07883	0.00005
SLC15A1	solute carrier family 15 member 1 [Source:NCBI gene;Acc:378789]	6.20921	0.740538	-3.06776	0.00045

ENSGALG00000019554	serpin peptidase inhibitor, clade B (ovalbumin), member 10 A [Source:NCBI gene;Acc:101749622]	6.96143	0.840034	-3.05086	0.0009
ENSGALG00000045596	OX-2 membrane glycoprotein-like [Source:NCBI gene;Acc:770026]	7.05759	0.857032	-3.04175	0.0002
ENSGALG00000037166	uncharacterized LOC101752158 [Source:NCBI gene;Acc:101752158]	15.534	1.89079	-3.03837	0.00005
ENSGALG00000010943	sodium voltage-gated channel alpha subunit 1 [Source:NCBI gene;Acc:771555]	2.41075	0.300052	-3.0062	0.00025
MMP1	matrix metallopeptidase 1 [Source:HGNC Symbol;Acc:HGNC:7155]	2.27122	0.287773	-2.98046	0.01075
ORM1	orosomucoid 1 (ovoglycoprotein) [Source:NCBI gene;Acc:395220]	12.9122	1.63605	-2.98045	0.0005
GNAT3	G protein subunit alpha transducin 3 [Source:NCBI gene;Acc:427851]	7.71618	0.980616	-2.97613	0.00185
DIO2	deiodinase, iodothyronine type II [Source:NCBI gene;Acc:373903]	28.423	3.68008	-2.94925	0.00005
HELZ2	helicase with zinc finger 2 [Source:HGNC Symbol;Acc:HGNC:30021]	372.728	48.2857	-2.94846	0.00015
KLHL10	kelch like family member 10 [Source:NCBI gene;Acc:420033]	18.7326	2.43064	-2.94614	0.00005
CALHM6	calcium homeostasis modulator family member 6 [Source:HGNC Symbol;Acc:HGNC:33391]	20.3239	2.64509	-2.94179	0.00005
ENSGALG00000045534	promyelocytic leukemia-like [Source:NCBI gene;Acc:100857563]	65.5315	8.57932	-2.93325	0.00005
OASL	2'-5'-oligoadenylate synthetase like [Source:NCBI gene;Acc:395908]	1653.76	225.94	-2.87174	0.00005
APOLD1	apolipoprotein L domain containing 1 [Source:HGNC Symbol;Acc:HGNC:25268]	30.7209	4.20289	-2.86977	0.00005
ENSGALG00000010032		1.95283	0.267763	-2.86654	0.00555
SPAM1	sperm adhesion molecule 1 [Source:HGNC Symbol;Acc:HGNC:11217]	3.39186	0.467133	-2.86017	0.00965
CLCA1	chloride channel accessory 1 [Source:HGNC Symbol;Acc:HGNC:2015]	1.70409	0.237003	-2.84602	0.00815
ENSGALG00000009479	sterile alpha motif domain containing 9 like [Source:NCBI gene;Acc:420559]	546.351	76.478	-2.83671	0.00005

ENSGALG00000054787	testis and ovary specific PAZ domain containing 1 [Source:NCBI gene;Acc:100857244]	2.03684	0.292471	-2.79997	0.0097
SAMD14	sterile alpha motif domain containing 14 [Source:HGNC Symbol;Acc:HGNC:27312]	3.92395	0.565957	-2.79354	0.00115
TGM4	transglutaminase 4 [Source:NCBI gene;Acc:420706]	56.1853	8.12057	-2.79054	0.00005
TEX12	testis expressed 12 [Source:HGNC Symbol;Acc:HGNC:11734]	1.47125	0.214813	-2.77589	0.0367
IFITM5	interferon induced transmembrane protein 5 [Source:NCBI gene;Acc:422992]	62.2775	9.17145	-2.76349	0.00005
THEMIS2	thymocyte selection associated family member 2 [Source:HGNC Symbol;Acc:HGNC:16839]	26.5855	3.95159	-2.75013	0.00005
GADD45G	growth arrest and DNA damage inducible gamma [Source:NCBI gene;Acc:429929]	45.0989	6.86267	-2.71625	0.00005
SLC25A47	solute carrier family 25 member 47 [Source:HGNC Symbol;Acc:HGNC:20115]	9.34195	1.42618	-2.71157	0.00365
CMPK2	cytidine/uridine monophosphate kinase 2 [Source:HGNC Symbol;Acc:HGNC:27015]	916.165	140.356	-2.70652	0.00005
IL8L1	interleukin 8-like 1 [Source:NCBI gene;Acc:395872]	2.43612	0.376575	-2.69358	0.01795
DHX58	DExH-box helicase 58 [Source:NCBI gene;Acc:100858653]	51.2436	8.02028	-2.67565	0.00005
NANP	N-acetylneuraminic acid phosphatase [Source:NCBI gene;Acc:421467]	97.5755	15.3177	-2.67132	0.02155
MX1	myxovirus (influenza virus) resistance 1, interferon-inducible protein p78 (mouse) [Source:NCBI gene;Acc:395313]	777.642	122.624	-2.66486	0.00005
ADAM8	ADAM metallopeptidase domain 8 [Source:HGNC Symbol;Acc:HGNC:215]	1.70062	0.271307	-2.64806	0.0085
PDE6B	phosphodiesterase 6B [Source:NCBI gene;Acc:395092]	1.32649	0.21374	-2.63368	0.01285
BATF3	basic leucine zipper ATF-like transcription factor 3 [Source:HGNC Symbol;Acc:HGNC:28915]	61.7572	9.96031	-2.63234	0.00005
IFI27L2	interferon, alpha-inducible protein 27-like 2 [Source:NCBI gene;Acc:403121]	3184.68	517.324	-2.62201	0.00005

ENSGALG00000005257	vascular cell adhesion molecule 1 [Source:NCBI gene;Acc:424467]	414.569	67.963	-2.60879	0.00005
ENSGALG00000007118	sodium/hydrogen exchanger 2-like [Source:NCBI gene;Acc:422276]	1.30231	0.214842	-2.59972	0.03965
AVD	avidin [Source:NCBI gene;Acc:396260]	924.545	152.708	-2.59797	0.00005
PPM1J	protein phosphatase, Mg2+/Mn2+ dependent 1J [Source:HGNC Symbol;Acc:HGNC:20785]	13.6068	2.25564	-2.59272	0.0003
B3GNT7	UDP-GlcNAc:betaGal beta-1,3-N-acetylglucosaminyltransferase 7 [Source:HGNC Symbol;Acc:HGNC:18811]	18.9794	3.14969	-2.59115	0.00005
ENSGALG00000047573		4.3303	0.719523	-2.58935	0.0041
ENSGALG00000011544	BCL2 like 14 [Source:NCBI gene;Acc:419096]	17.87	2.97394	-2.58709	0.00005
SLBP	stem-loop binding protein [Source:NCBI gene;Acc:422902]	108.927	18.1593	-2.58458	0.00005
PMAIP1	phorbol-12-myristate-13-acetate-induced protein 1 [Source:NCBI gene;Acc:770077]	67.8451	11.3235	-2.58292	0.00005
RNF19B	ring finger protein 19B [Source:HGNC Symbol;Acc:HGNC:26886]	61.3465	10.343	-2.56833	0.00005
IFIH1	interferon induced with helicase C domain 1 [Source:NCBI gene;Acc:424185]	451.023	76.7212	-2.5555	0.00005
STOML1	stomatin like 1 [Source:NCBI gene;Acc:415303]	82.8888	14.196	-2.54569	0.00005
ATF3	activating transcription factor 3 [Source:HGNC Symbol;Acc:HGNC:785]	33.9602	5.87454	-2.5313	0.01995
ENSGALG00000015032	CD274 molecule [Source:NCBI gene;Acc:427224]	121.871	21.0937	-2.53047	0.00005
ENSGALG00000031430	selectin E [Source:NCBI gene;Acc:424402]	93.5701	16.2871	-2.52232	0.00005
DDIT4	DNA damage inducible transcript 4 [Source:HGNC Symbol;Acc:HGNC:24944]	50.7887	8.86814	-2.5178	0.02695
ENSGALG00000013057	ubiquitin specific peptidase 41 [Source:NCBI gene;Acc:418167]	537.367	93.9026	-2.51667	0.00005
OGFR	opioid growth factor receptor [Source:HGNC Symbol;Acc:HGNC:15768]	80.7789	14.1329	-2.51492	0.00005
PTX3	pentraxin 3 [Source:NCBI gene;Acc:548626]	1.88888	0.330477	-2.51491	0.00945
ENSGALG00000019147	granulocyte-macrophage colony-stimulating factor receptor	55.0708	9.68182	-2.50794	0.00005

	subunit alpha-like [Source:NCBI gene;Acc:418667]				
DPEP2	dipeptidase 2 [Source:HGNC Symbol;Acc:HGNC:23028]	45.0743	7.93338	-2.5063	0.00005
ENSGALG00000042001	hect domain and RLD 4-like [Source:NCBI gene;Acc:422513]	425.699	75.1196	-2.50257	0.00005
CMTR1	cap methyltransferase 1 [Source:HGNC Symbol;Acc:HGNC:21077]	242.015	42.7081	-2.50251	0.00005
GCH1	GTP cyclohydrolase 1 [Source:NCBI gene;Acc:396146]	30.5171	5.51034	-2.4694	0.00005
GBP	guanylate binding protein [Source:NCBI gene;Acc:395366]	64.3969	11.6318	-2.46892	0.00005
ENSGALG00000016964		265.304	48.2609	-2.45872	0.00005
ENSGALG00000051554		5.69598	1.04379	-2.44811	0.0029
ATP6V0D2	ATPase H+ transporting V0 subunit d2 [Source:NCBI gene;Acc:420210]	29.6145	5.45703	-2.44012	0.00005
CYP2W1	cytochrome P450 family 2 subfamily W member 1 [Source:HGNC Symbol;Acc:HGNC:20243]	3.35937	0.620552	-2.43657	0.00145
ENSGALG00000007123	uncharacterized LOC101750621 [Source:NCBI gene;Acc:101750621]	16.4805	3.09518	-2.41266	0.00005
NCF2	neutrophil cytosolic factor 2 [Source:HGNC Symbol;Acc:HGNC:7661]	4.93484	0.9355	-2.39919	0.0002
ENSGALG00000053042		9.17641	1.74042	-2.39849	0.0107
ANGPT1	angiopoietin 1 [Source:NCBI gene;Acc:395129]	68.4093	13.0773	-2.38713	0.00005
SOCS3	suppressor of cytokine signaling 3 [Source:NCBI gene;Acc:395299]	61.8441	11.8482	-2.38397	0.00005
IFI6	interferon alpha inducible protein 6 [Source:NCBI gene;Acc:403120]	735.354	141.01	-2.38264	0.00005
CXorf21	chromosome X open reading frame 21 [Source:HGNC Symbol;Acc:HGNC:25667]	10.243	1.96726	-2.38038	0.00015
EIF2AK2	eukaryotic translation initiation factor 2 alpha kinase 2 [Source:NCBI gene;Acc:395147]	327.546	64.005	-2.35544	0.00005
DDX60	DExD/H-box helicase 60 [Source:HGNC Symbol;Acc:HGNC:25942]	320.005	63.186	-2.34042	0.00005
IL22RA2	interleukin 22 receptor subunit alpha 2 [Source:HGNC Symbol;Acc:HGNC:14901]	10.1272	2.04242	-2.30988	0.00005

P2RX7	purinergic receptor P2X 7 [Source:HGNC Symbol;Acc:HGNC:8537]	189.203	38.224	-2.30738	0.00005
NT5C3B	5'-nucleotidase, cytosolic IIIB [Source:NCBI gene;Acc:420034]	88.5782	18.1385	-2.2879	0.00005
TLR3	toll like receptor 3 [Source:NCBI gene;Acc:422720]	113.433	23.2434	-2.28695	0.00005
ENSGALG00000026299		10.0827	2.0887	-2.2712	0.0002
HMOX1	heme oxygenase 1 [Source:NCBI gene;Acc:396287]	43.25	8.9929	-2.26584	0.00355
ZC3HAV1	zinc finger CCCH-type containing, antiviral 1 [Source:NCBI gene;Acc:426315]	214.851	44.8193	-2.26114	0.00015
ENSGALG00000014585	C-C motif chemokine ligand 26 [Source:NCBI gene;Acc:417534]	376.294	78.7221	-2.25702	0.00005
ENSGALG00000046098	ubiquitin-conjugating enzyme E2L 6 [Source:NCBI gene;Acc:100858073]	170.019	35.6728	-2.2528	0.00975
CD200L	CD200 molecule-like [Source:NCBI gene;Acc:418424]	1.95603	0.411588	-2.24866	0.0153
LIPA	lipase A, lysosomal acid type [Source:HGNC Symbol;Acc:HGNC:6617]	506.614	107.103	-2.24189	0.00005
ISL2	ISL LIM homeobox 2 [Source:HGNC Symbol;Acc:HGNC:18524]	3.4146	0.723001	-2.23965	0.03345
ETV7	ETS variant 7 [Source:HGNC Symbol;Acc:HGNC:18160]	10.0219	2.12446	-2.23799	0.00165
IRF9	interferon regulatory factor 9 [Source:NCBI gene;Acc:395243]	36.153	7.66937	-2.23694	0.00005
MSC	musculin [Source:HGNC Symbol;Acc:HGNC:7321]	8.2048	1.7421	-2.23564	0.00875
ENSGALG00000015662	lipase member I [Source:NCBI gene;Acc:418466]	22.2884	4.74717	-2.23115	0.0002
ENSGALG00000028016	eye-globin [Source:NCBI gene;Acc:417972]	42.0567	8.98146	-2.22731	0.00005
ENSGALG00000004243	interferon-induced transmembrane protein 3-like [Source:NCBI gene;Acc:422993]	66.1984	14.1404	-2.22697	0.0002
PARP9	poly(ADP-ribose) polymerase family member 9 [Source:HGNC Symbol;Acc:HGNC:24118]	265.1	56.7261	-2.22445	0.0003
ENSGALG00000040131	calcium/calmodulin dependent protein kinase kinase 2 [Source:NCBI gene;Acc:396405]	58.6903	12.5845	-2.22147	0.00005
ENSGALG00000021399	ATP binding cassette subfamily A member 8 [Source:NCBI gene;Acc:417440]	21.804	4.74037	-2.20152	0.00005

CSF2RA	colony stimulating factor 2 receptor alpha subunit [Source:NCBI gene;Acc:418666]	45.7014	9.94109	-2.20076	0.00005
CRISPLD2	cysteine rich secretory protein LCCL domain containing 2 [Source:HGNC Symbol;Acc:HGNC:25248]	70.0924	15.2792	-2.19769	0.00005
ACOD1	aconitate decarboxylase 1 [Source:NCBI gene;Acc:418812]	32.731	7.13803	-2.19706	0.00595
ENSGALG00000030571		119.46	26.0803	-2.1955	0.00005
IFI35	interferon induced protein 35 [Source:HGNC Symbol;Acc:HGNC:5399]	153.932	33.6304	-2.19446	0.001
STAT1	signal transducer and activator of transcription 1 [Source:NCBI gene;Acc:424044]	895.387	195.632	-2.19437	0.00005
TRAFD1	TRAF-type zinc finger domain containing 1 [Source:NCBI gene;Acc:416884]	156.241	34.3221	-2.18656	0.00005
CXCR1	C-X-C motif chemokine receptor 1 [Source:NCBI gene;Acc:430652]	10.3537	2.28378	-2.18065	0.001
ATP8A1	ATPase phospholipid transporting 8A1 [Source:HGNC Symbol;Acc:HGNC:13531]	211.881	46.7965	-2.17878	0.00005
NMI	N-myc and STAT interactor [Source:HGNC Symbol;Acc:HGNC:7854]	406.554	89.7951	-2.17874	0.00005
PIGA	phosphatidylinositol glycan anchor biosynthesis class A [Source:NCBI gene;Acc:418624]	38.7042	8.56251	-2.17638	0.00005
ENSGALG00000032428	chromosome 11 open reading frame, human C19orf12-like [Source:NCBI gene;Acc:100858381]	756.975	167.966	-2.17208	0.00035
ENSGALG00000026152	guanylate-binding protein 4-like [Source:NCBI gene;Acc:415922]	639.768	142.686	-2.16471	0.00005
CCL17	C-C motif chemokine ligand 17 [Source:NCBI gene;Acc:415652]	16.3108	3.64513	-2.16179	0.00505
CRHR2	corticotropin releasing hormone receptor 2 [Source:NCBI gene;Acc:395105]	4.70544	1.05168	-2.16163	0.0013
RLN3	relaxin 3 [Source:NCBI gene;Acc:427223]	15.2099	3.4106	-2.15691	0.00245
SNX10	sorting nexin 10 [Source:NCBI gene;Acc:420628]	135.793	30.4726	-2.15582	0.00005
COL9A3	collagen type IX alpha 3 chain [Source:NCBI gene;Acc:396242]	8.77888	1.98448	-2.14528	0.00005

CEBPD	CCAAT enhancer binding protein delta [Source:HGNC Symbol;Acc:HGNC:1835]	167.053	37.8501	-2.14194	0.00005
ENSGALG00000049067	regulating synaptic membrane exocytosis 1 [Source:NCBI gene;Acc:421867]	9.1936	2.08495	-2.14062	0.00355
MINDY3	MINDY lysine 48 deubiquitinase 3 [Source:HGNC Symbol;Acc:HGNC:23578]	142.773	32.4354	-2.13808	0.00005
TNIP3	TNFAIP3 interacting protein 3 [Source:HGNC Symbol;Acc:HGNC:19315]	119.269	27.1138	-2.13712	0.00005
ENSGALG00000015692	DNA polymerase nu [Source:NCBI gene;Acc:422892]	1.35746	0.309236	-2.13413	0.04295
PHACTR3	phosphatase and actin regulator 3 [Source:HGNC Symbol;Acc:HGNC:15833]	4.32574	0.992599	-2.12366	0.00105
ENSGALG00000001304	transmembrane protein 52 [Source:NCBI gene;Acc:419401]	22.0024	5.07607	-2.11588	0.00005
GKAP1	G kinase anchoring protein 1 [Source:HGNC Symbol;Acc:HGNC:17496]	53.2477	12.4182	-2.10026	0.00005
TMEM173	transmembrane protein 173 [Source:NCBI gene;Acc:768990]	29.7459	6.94309	-2.09904	0.00005
NAMPTP1	nicotinamide phosphoribosyltransferase pseudogene 1 [Source:NCBI gene;Acc:417707]	196.882	45.9758	-2.09838	0.00005
RGS1	regulator of G protein signaling 1 [Source:HGNC Symbol;Acc:HGNC:9991]	23.0825	5.40576	-2.09423	0.00005
GGT5	gamma-glutamyltransferase 5 [Source:HGNC Symbol;Acc:HGNC:4260]	152.025	35.9338	-2.0809	0.00005
ENSGALG00000011324	tubulin alpha 4b [Source:NCBI gene;Acc:100857214]	10.0643	2.38012	-2.08014	0.00015
IL8	interleukin 8-like 2 [Source:NCBI gene;Acc:396495]	28.0238	6.65054	-2.07511	0.0001
HTR7	5-hydroxytryptamine (serotonin) receptor 7, adenylate cyclase-coupled [Source:NCBI gene;Acc:423794]	20.9027	4.96524	-2.07375	0.0005
GLUL	glutamate-ammonia ligase [Source:NCBI gene;Acc:396489]	82.8549	19.9412	-2.05483	0.00005
ENSGALG00000015822	MAP3K7 C-terminal like [Source:NCBI gene;Acc:418487]	3.25392	0.788402	-2.04518	0.01615
CCL19	C-C motif chemokine ligand 19 [Source:HGNC Symbol;Acc:HGNC:10617]	176.984	42.9061	-2.04436	0.0001

SHISA3	shisa family member 3 [Source:HGNC Symbol;Acc:HGNC:25159]	2.47308	0.600942	-2.04101	0.0189 5
ENSGALG00000035 453	retinitis pigmentosa 9 (autosomal dominant) [Source:NCBI gene;Acc:420747]	64.3468	15.6689	-2.03796	0.0000 5
BHLHE22	basic helix-loop-helix family member e22 [Source:NCBI gene;Acc:395164]	3.15477	0.774148	-2.02685	0.0118 5
RSFR	leukocyte ribonuclease A-2 [Source:NCBI gene;Acc:423668]	576.507	142.454	-2.01684	0.0001 5
ENSGALG00000048 671	E3 ubiquitin/ISG15 ligase TRIM25-like [Source:NCBI gene;Acc:416147]	261.155	65.4033	-1.99747	0.0000 5
EDN2	endothelin 2 [Source:NCBI gene;Acc:419559]	13.9962	3.52281	-1.99024	0.0000 5
C1S	complement C1s [Source:NCBI gene;Acc:418294]	214.844	54.6317	-1.97548	0.0000 5
STAT2	signal transducer and activator of transcription 2 [Source:HGNC Symbol;Acc:HGNC:11363]	81.1847	20.7059	-1.97117	0.0000 5
K123	K123 protein [Source:NCBI gene;Acc:395873]	16.5284	4.23885	-1.9632	0.0000 5
ENSGALG00000053 794		24.7283	6.35281	-1.9607	0.0005
ENSGALG00000046 593	adrenomedullin [Source:NCBI gene;Acc:423042]	33.5756	8.62973	-1.96003	0.0383 5
ENSGALG00000044 816		15.2174	3.91229	-1.95964	0.0017 5
ENSGALG00000011 806	mucin 13, cell surface associated [Source:NCBI gene;Acc:100859223]	179.755	46.2964	-1.95706	0.0000 5
PHF11	PHD finger protein 11 [Source:HGNC Symbol;Acc:HGNC:17024]	80.9804	20.9021	-1.95392	0.0000 5
ENSGALG00000039 727		6.95496	1.80009	-1.94997	0.0009 5
PLS1	plastin 1 [Source:NCBI gene;Acc:396291]	3.82738	0.992313	-1.94749	0.0026
ENSGALG00000026 422	poly(ADP-ribose) polymerase family member 14 [Source:NCBI gene;Acc:101747378]	224.95	58.6154	-1.94025	0.0000 5
ENSGALG00000047 412	poly [ADP-ribose] polymerase 10-like [Source:NCBI gene;Acc:112530987]	19.2552	5.02192	-1.93894	0.0011
IL1R2	interleukin 1 receptor type 2 [Source:HGNC Symbol;Acc:HGNC:5994]	16.929	4.4277	-1.93487	0.0000 5
IRF5	interferon regulatory factor 5 [Source:NCBI gene;Acc:430409]	9.38458	2.46369	-1.92947	0.0000 5

DNAJC13	DnaJ heat shock protein family (Hsp40) member C13 [Source:HGNC Symbol;Acc:HGNC:30343]	118.575	31.171	-1.92752	0.00005
ENSGALG00000049551	chemokine [Source:NCBI gene;Acc:417535]	16.8408	4.43817	-1.92392	0.00445
C11H19orf12	chromosome 11 C19orf12 homolog [Source:NCBI gene;Acc:415757]	211.994	56.1972	-1.91545	0.03945
RNF24	ring finger protein 24 [Source:HGNC Symbol;Acc:HGNC:13779]	34.8296	9.236	-1.91497	0.00005
VTG1	vitellogenin 1 [Source:NCBI gene;Acc:424547]	3.0715	0.816231	-1.91189	0.00015
TMCC3	transmembrane and coiled-coil domain family 3 [Source:HGNC Symbol;Acc:HGNC:29199]	33.4489	8.9096	-1.90853	0.00005
PDXK	pyridoxal (pyridoxine, vitamin B6) kinase [Source:NCBI gene;Acc:418549]	148.501	39.5929	-1.90716	0.00005
CPS1	carbamoyl-phosphate synthase 1 [Source:NCBI gene;Acc:428994]	6.1987	1.65591	-1.90434	0.00005
ZBTB32	zinc finger and BTB domain containing 32 [Source:NCBI gene;Acc:419759]	73.6913	19.7115	-1.90246	0.00005
IL1RL1	interleukin 1 receptor like 1 [Source:NCBI gene;Acc:374136]	48.686	13.0242	-1.90231	0.00005
BANK1	B cell scaffold protein with ankyrin repeats 1 [Source:HGNC Symbol;Acc:HGNC:18233]	2.81711	0.755856	-1.89803	0.00265
ENSGALG00000012021	potassium channel, subfamily K, member 4 [Source:NCBI gene;Acc:423536]	52.5165	14.1833	-1.88858	0.00005
RNF213	ring finger protein 213 [Source:HGNC Symbol;Acc:HGNC:14539]	465.179	125.761	-1.8871	0.0086
ENSGALG00000019755		11.4509	3.10167	-1.88434	0.001
C1R	complement C1r [Source:HGNC Symbol;Acc:HGNC:1246]	64.8029	17.5543	-1.88423	0.00005
ENSGALG00000015944		3.56214	0.969077	-1.87806	0.0105
BAIAP2L2	BAI1 associated protein 2 like 2 [Source:HGNC Symbol;Acc:HGNC:26203]	2.72191	0.744547	-1.87018	0.008
RNF144B	ring finger protein 144B [Source:HGNC Symbol;Acc:HGNC:21578]	36.6947	10.0473	-1.86876	0.00005
LY96	lymphocyte antigen 96 [Source:HGNC Symbol;Acc:HGNC:17156]	82.7482	22.6625	-1.86842	0.00005

PAPSS2	3'-phosphoadenosine 5'-phosphosulfate synthase 2 [Source:HGNC Symbol;Acc:HGNC:8604]	7.99654	2.19481	-1.86528	0.0008
IGF2BP3	insulin like growth factor 2 mRNA binding protein 3 [Source:NCBI gene;Acc:420617]	49.8582	13.7455	-1.85887	0.00005
CYSLTR2	cysteinyl leukotriene receptor 2 [Source:HGNC Symbol;Acc:HGNC:18274]	12.4001	3.42834	-1.85477	0.00005
ASPA	aspartoacylase [Source:HGNC Symbol;Acc:HGNC:756]	12.9539	3.58415	-1.85368	0.00105
ADAP2	ArfGAP with dual PH domains 2 [Source:HGNC Symbol;Acc:HGNC:16487]	16.86	4.67987	-1.84906	0.00005
TLR15	Gallus gallus toll-like receptor 15 (TLR15), mRNA. [Source:RefSeq mRNA;Acc:NM_001037835]	8.46721	2.37059	-1.83664	0.00025
ENSGALG00000045090	osteoclast-associated immunoglobulin-like receptor-like [Source:NCBI gene;Acc:101750289]	2.10426	0.589449	-1.83587	0.0342
MYD88	myeloid differentiation primary response 88 [Source:NCBI gene;Acc:420420]	161.688	45.3904	-1.83275	0.00005
ENSGALG00000003644	C2 calcium dependent domain containing 4A [Source:NCBI gene;Acc:427491]	5.38633	1.51495	-1.83003	0.0073
IL18	interleukin 18 [Source:NCBI gene;Acc:395312]	77.629	21.9579	-1.82186	0.0002
DUSP4	dual specificity phosphatase 4 [Source:NCBI gene;Acc:395642]	4.66504	1.32207	-1.81909	0.0077
ARID5A	AT-rich interaction domain 5A [Source:HGNC Symbol;Acc:HGNC:17361]	31.27	8.87421	-1.81709	0.00005
NBN	nibrin [Source:NCBI gene;Acc:374246]	68.5193	19.4495	-1.81678	0.00005
GADD45B	growth arrest and DNA damage inducible beta [Source:HGNC Symbol;Acc:HGNC:4096]	36.1389	10.3268	-1.80716	0.00005
TTC31	tetratricopeptide repeat domain 31 [Source:HGNC Symbol;Acc:HGNC:25759]	70.759	20.2583	-1.8044	0.00005
TTPA	alpha tocopherol transfer protein [Source:HGNC Symbol;Acc:HGNC:12404]	7.84605	2.25135	-1.80118	0.0145
ENSGALG00000004113	putative P2Y purinoceptor 10-like [Source:NCBI gene;Acc:422147]	14.8175	4.26394	-1.79704	0.00005

DGLUCY	D-glutamate cyclase [Source:HGNC Symbol;Acc:HGNC:20498]	4.73205	1.36474	-1.79384	0.0021
ENSGALG00000042500		6.5479	1.89181	-1.79127	0.00505
ENSGALG00000034756	connective tissue growth factor-like [Source:NCBI gene;Acc:776271]	1.81581	0.526486	-1.78615	0.0037
ENSGALG00000027587	leucine rich alpha-2-glycoprotein 1 [Source:NCBI gene;Acc:100858066]	14.9789	4.34674	-1.78493	0.0003
IRF7	interferon regulatory factor 7 [Source:NCBI gene;Acc:396330]	134.103	38.9849	-1.78235	0.00005
CHRD	chordin [Source:NCBI gene;Acc:395828]	4.97637	1.44762	-1.78141	0.0001
AOX1	aldehyde oxidase 1 [Source:NCBI gene;Acc:424071]	13.6159	3.97172	-1.77746	0.00005
ENSGALG00000032066		3.28785	0.9694	-1.76198	0.04875
APOA1	apolipoprotein A1 [Source:NCBI gene;Acc:396536]	709.841	209.619	-1.75973	0.0004
GCG	glucagon [Source:NCBI gene;Acc:396196]	9.12327	2.71706	-1.7475	0.00445
ENSGALG00000027778	ras-related and estrogen-regulated growth inhibitor-like [Source:NCBI gene;Acc:770492]	5.37417	1.60422	-1.74417	0.00475
ENSGALG00000050946	uncharacterized LOC426064 [Source:NCBI gene;Acc:426064]	176.388	52.6554	-1.7441	0.00005
ENSGALG00000045478	exocyst complex component 3 like [Source:NCBI gene;Acc:423478]	45.5001	13.6321	-1.73886	0.00005
ZNFX1	zinc finger NFX1-type containing 1 [Source:HGNC Symbol;Acc:HGNC:29271]	433.65	130.092	-1.737	0.0002
IL1B	interleukin 1, beta [Source:NCBI gene;Acc:395196]	3.10319	0.931864	-1.73556	0.02475
ENSGALG00000047283	deleted in malignant brain tumors 1 [Source:NCBI gene;Acc:426819]	68.8577	20.7777	-1.72858	0.00005
TNFRSF11B	TNF receptor superfamily member 11b [Source:NCBI gene;Acc:378803]	73.1837	22.121	-1.72611	0.00005
SLC2A6	solute carrier family 2 member 6 [Source:HGNC Symbol;Acc:HGNC:11011]	20.852	6.30878	-1.72475	0.00005
ENSGALG00000051980		27.3215	8.31283	-1.71663	0.00005
ENSGALG00000053705		21.889	6.70617	-1.70665	0.0234

TESMIN	testis expressed metallothionein like protein [Source:HGNC Symbol;Acc:HGNC:7446]	1.84938	0.570201	-1.6975	0.03175
SEMA3F	semaphorin 3F [Source:NCBI gene;Acc:374109]	15.0328	4.63575	-1.69724	0.00005
ENSGALG00000045199	dispanin subfamily A member 2b-like [Source:NCBI gene;Acc:107053353]	1737.16	536.685	-1.69458	0.00005
TRIM25	tripartite motif containing 25 [Source:NCBI gene;Acc:417401]	215.982	66.8328	-1.69228	0.00005
RCAN2	regulator of calcineurin 2 [Source:HGNC Symbol;Acc:HGNC:3041]	3.89659	1.20853	-1.68896	0.0415
SUCNR1	succinate receptor 1 [Source:HGNC Symbol;Acc:HGNC:4542]	11.2619	3.50285	-1.68485	0.0015
IL13RA2	interleukin 13 receptor subunit alpha 2 [Source:NCBI gene;Acc:422219]	5.7236	1.78158	-1.68377	0.0027
ENSGALG00000019276	solute carrier organic anion transporter family member 1B1 [Source:NCBI gene;Acc:418189]	9.49485	2.96822	-1.67755	0.0002
SPIRE2	spire type actin nucleation factor 2 [Source:HGNC Symbol;Acc:HGNC:30623]	4.16936	1.30744	-1.67308	0.0031
ENSGALG00000025881	colony stimulating factor 2 receptor beta common subunit [Source:NCBI gene;Acc:101750812]	26.9215	8.45183	-1.67142	0.00415
APAF1	apoptotic peptidase activating factor 1 [Source:HGNC Symbol;Acc:HGNC:576]	96.7243	30.4562	-1.66714	0.00005
USP25	ubiquitin specific peptidase 25 [Source:HGNC Symbol;Acc:HGNC:12624]	153.221	48.263	-1.66662	0.00005
ASPG	asparaginase [Source:HGNC Symbol;Acc:HGNC:20123]	19.7759	6.23695	-1.66483	0.00005
SPI1	Spi-1 proto-oncogene [Source:NCBI gene;Acc:395879]	217.463	68.6747	-1.66292	0.0001
ENSGALG00000012072	poly(ADP-ribose) polymerase family member 14 [Source:NCBI gene;Acc:101747378]	574.526	181.706	-1.66077	0.00075
COMMD8	COMM domain containing 8 [Source:NCBI gene;Acc:422769]	90.3671	28.597	-1.65993	0.00005
OTUD4	OTU deubiquitinase 4 [Source:HGNC Symbol;Acc:HGNC:24949]	112.865	35.8704	-1.65373	0.00005
EFHD1	EF-hand domain family member D1 [Source:NCBI gene;Acc:424741]	169.304	53.9568	-1.64974	0.00005

CD1B	CD1b molecule [Source:NCBI gene;Acc:425802]	21.3885	6.83965	-1.64484	0.0001
MARCO	macrophage receptor with collagenous structure [Source:NCBI gene;Acc:395488]	49.1118	15.7141	-1.64401	0.00005
MT3	metallothionein 3 [Source:NCBI gene;Acc:770592]	39.0324	12.5006	-1.64267	0.005
S100A16	S100 calcium binding protein A16 [Source:HGNC Symbol;Acc:HGNC:20441]	18.4963	5.94206	-1.6382	0.02905
SMC6	structural maintenance of chromosomes 6 [Source:NCBI gene;Acc:421950]	49.2022	15.8114	-1.63776	0.0002
BCO1	Gallus gallus beta-carotene oxygenase 1 (BCO1), mRNA. [Source:RefSeq mRNA;Acc:NM_001364902]	62.655	20.1853	-1.63412	0.00005
ENSGALG00000047545		3.12963	1.01254	-1.62801	0.00535
ENSGALG00000027407	G protein-coupled receptor 132 [Source:NCBI gene;Acc:100859188]	21.8499	7.06962	-1.62792	0.00005
DTX3L	deltex E3 ubiquitin ligase 3L [Source:HGNC Symbol;Acc:HGNC:30323]	221.225	71.7064	-1.62534	0.0146
IKBIP	IKBKB interacting protein [Source:HGNC Symbol;Acc:HGNC:26430]	52.6011	17.1043	-1.62073	0.00005
SDC4	syndecan 4 [Source:NCBI gene;Acc:419184]	220.021	71.7627	-1.61634	0.00005
ENSGALG00000011872	NFAT activating protein with ITAM motif 1 [Source:NCBI gene;Acc:417979]	17.3552	5.6618	-1.61604	0.00005
PLSCR1	phospholipid scramblase 1 [Source:HGNC Symbol;Acc:HGNC:9092]	305.5	99.8406	-1.61347	0.00005
C1QC	complement C1q C chain [Source:HGNC Symbol;Acc:HGNC:1245]	118.818	38.8847	-1.61148	0.00005
TSPAN8	tetraspanin 8 [Source:NCBI gene;Acc:417854]	4.54466	1.48997	-1.60889	0.0321
SIRPA	signal-regulatory protein alpha [Source:NCBI gene;Acc:419267]	5.79938	1.90152	-1.60875	0.04665
ETV4	ETS variant 4 [Source:HGNC Symbol;Acc:HGNC:3493]	4.30828	1.41429	-1.60703	0.00965
ENSGALG00000040120	glycine N-acyltransferase-like protein 3 [Source:NCBI gene;Acc:107049269]	9.0542	2.97671	-1.60487	0.0304
ENSGALG00000044326	deleted in malignant brain tumors 1 protein-like [Source:NCBI gene;Acc:426820]	53.5008	17.5954	-1.60436	0.00005

ENSGALG00000007268	calcium dependent secretion activator [Source:NCBI gene;Acc:416075]	4.15153	1.37373	-1.59554	0.0011
TUT4	terminal uridylyl transferase 4 [Source:HGNC Symbol;Acc:HGNC:28981]	136.681	45.2837	-1.59375	0.00005
ABI3	ABI family member 3 [Source:HGNC Symbol;Acc:HGNC:29859]	9.46327	3.13541	-1.59368	0.00035
FOS	Fos proto-oncogene, AP-1 transcription factor subunit [Source:NCBI gene;Acc:396512]	105.261	34.9906	-1.58893	0.00005
ENSGALG00000016556	GTPase, very large interferon inducible pseudogene 1 [Source:NCBI gene;Acc:428660]	81.451	27.1158	-1.5868	0.00005
ADAMTS1	ADAM metallopeptidase with thrombospondin type 1 motif 1 [Source:HGNC Symbol;Acc:HGNC:217]	43.2223	14.4376	-1.58194	0.00005
SOX14	SRY-box 14 [Source:NCBI gene;Acc:395526]	1.82445	0.6098	-1.58105	0.04775
MARCH1	membrane associated ring-CH-type finger 1 [Source:NCBI gene;Acc:422420]	34.5667	11.6051	-1.57462	0.00005
RHOC	ras homolog family member C [Source:NCBI gene;Acc:395869]	19.8161	6.65425	-1.57433	0.00035
OR51E2	olfactory receptor family 51 subfamily E member 2 [Source:HGNC Symbol;Acc:HGNC:15195]	8.20585	2.76818	-1.56772	0.0208
ENSGALG00000040269		9.06806	3.05947	-1.56751	0.01705
ENSGALG00000026970	interferon-induced transmembrane protein 1-like [Source:NCBI gene;Acc:770612]	5205.53	1757.08	-1.56687	0.00325
ELMO3	engulfment and cell motility 3 [Source:NCBI gene;Acc:100857533]	11.8167	3.9941	-1.56488	0.00005
MC2R	melanocortin 2 receptor [Source:NCBI gene;Acc:428516]	17.9667	6.07484	-1.56441	0.00215
ENSGALG00000045671	uncharacterized LOC416755 [Source:NCBI gene;Acc:416755]	7.93295	2.68918	-1.56069	0.0001
TNIP2	TNFAIP3 interacting protein 2 [Source:NCBI gene;Acc:422884]	16.4143	5.57372	-1.55824	0.00015
KAZN	kazrin, periplakin interacting protein [Source:HGNC Symbol;Acc:HGNC:29173]	25.1088	8.54183	-1.55558	0.00005
WDFY2	WD repeat and FYVE domain containing 2 [Source:NCBI gene;Acc:770107]	38.0545	12.9612	-1.55387	0.00005

CEBPB	CCAAT/enhancer binding protein beta [Source:NCBI gene;Acc:396185]	81.6441	27.8503	-1.55166	0.00005
PARP12	poly(ADP-ribose) polymerase family member 12 [Source:HGNC Symbol;Acc:HGNC:21919]	410.494	140.591	-1.54586	0.00015
ENSGALG00000051068		34.027	11.6859	-1.54191	0.00005
SLC25A29	solute carrier family 25 member 29 [Source:HGNC Symbol;Acc:HGNC:20116]	199.639	68.8251	-1.53639	0.00005
FCER1G	Fc fragment of IgE receptor Ig [Source:NCBI gene;Acc:100049618]	177.395	61.3343	-1.5322	0.0016
KCNMB1	potassium calcium-activated channel subfamily M regulatory beta subunit 1 [Source:NCBI gene;Acc:395301]	44.1795	15.3068	-1.5292	0.00005
C1QB	complement C1q B chain [Source:HGNC Symbol;Acc:HGNC:1242]	75.38	26.1773	-1.52587	0.00005
SAMHD1	SAM and HD domain containing deoxynucleoside triphosphate triphosphohydrolase 1 [Source:NCBI gene;Acc:419125]	204.488	71.0583	-1.52494	0.00005
PGF	placental growth factor [Source:HGNC Symbol;Acc:HGNC:8893]	4.32747	1.50528	-1.52349	0.0184
TMEM168	transmembrane protein 168 [Source:HGNC Symbol;Acc:HGNC:25826]	75.4518	26.2532	-1.52306	0.00005
ENSGALG00000009387	synaptojanin 2 binding protein [Source:NCBI gene;Acc:430724]	8.70865	3.03481	-1.52084	0.00075
MAFF	MAF bZIP transcription factor F [Source:NCBI gene;Acc:395519]	23.9891	8.36656	-1.51967	0.00005
ASS1	argininosuccinate synthase 1 [Source:NCBI gene;Acc:417185]	184.827	64.5075	-1.51864	0.00005
PRR5	proline rich 5 [Source:NCBI gene;Acc:418237]	11.9625	4.17713	-1.51793	0.00105
SIK1	salt inducible kinase 1 [Source:NCBI gene;Acc:395417]	69.164	24.181	-1.51615	0.00005
MAFB	MAF bZIP transcription factor B [Source:NCBI gene;Acc:419173]	43.7577	15.3046	-1.51557	0.00005
CYR61	cysteine rich angiogenic inducer 61 [Source:NCBI gene;Acc:429089]	85.2315	29.829	-1.51467	0.00005
FAU	FAU ubiquitin like and ribosomal protein S30 fusion [Source:HGNC Symbol;Acc:HGNC:3597]	64.5086	22.6223	-1.51175	0.02655

RAB40B	RAB40B, member RAS oncogene family [Source:NCBI gene;Acc:417338]	10.3821	3.64196	-1.51131	0.002
PTPRR	protein tyrosine phosphatase receptor type R [Source:HGNC Symbol;Acc:HGNC:9680]	7.28564	2.55872	-1.50963	0.00255
CNP	2',3'-cyclic nucleotide 3' phosphodiesterase [Source:NCBI gene;Acc:395921]	120.991	42.5193	-1.50871	0.00045
CD40	CD40 molecule [Source:NCBI gene;Acc:395385]	8.94658	3.14748	-1.50714	0.00205
LRRC15	leucine rich repeat containing 15 [Source:HGNC Symbol;Acc:HGNC:20818]	50.4434	17.7481	-1.507	0.00005
ENSGALG00000049433	platelet glycoprotein VI-like [Source:NCBI gene;Acc:112531135]	1.34054	0.47261	-1.50409	0.02335
HRH1	histamine receptor H1 [Source:HGNC Symbol;Acc:HGNC:5182]	12.4166	4.37754	-1.50408	0.0023
APOF	apolipoprotein F [Source:HGNC Symbol;Acc:HGNC:615]	8.53701	3.01946	-1.49944	0.03255
ENSGALG00000029446	transmembrane protein 110-like [Source:NCBI gene;Acc:419112]	30.7504	10.8997	-1.49632	0.00005
ENSGALG00000054321		1.40116	0.496848	-1.49575	0.0243
ENSGALG00000039708	PML-RARA regulated adaptor molecule 1 [Source:NCBI gene;Acc:420067]	11.1357	3.96881	-1.48841	0.0038
ENSGALG00000038146	tripartite motif containing 58 [Source:NCBI gene;Acc:770718]	42.095	15.008	-1.48792	0.0006
MAPK11	mitogen-activated protein kinase 11 [Source:NCBI gene;Acc:417739]	24.5062	8.74082	-1.48731	0.00245
BCL2A1	BCL2 related protein A1 [Source:NCBI gene;Acc:395673]	77.8059	27.7933	-1.48514	0.00005
ITGB6	integrin subunit beta 6 [Source:HGNC Symbol;Acc:HGNC:6161]	92.2814	32.9799	-1.48445	0.00005
ENSGALG00000016471	5'-nucleotidase, cytosolic IB [Source:NCBI gene;Acc:421954]	67.613	24.1891	-1.48294	0.00005
ENSGALG00000029066	discoidin, CUB and LCCL domain-containing protein 1-like [Source:NCBI gene;Acc:419584]	26.8647	9.6419	-1.47832	0.0378
STARD5	StAR related lipid transfer domain containing 5 [Source:HGNC Symbol;Acc:HGNC:18065]	64.0325	22.996	-1.47742	0.00005
MT4	metallothionein 4 [Source:NCBI gene;Acc:396212]	587.83	211.162	-1.47705	0.00005

CRLF2	cytokine receptor like factor 2 [Source:HGNC Symbol;Acc:HGNC:14281]	12.035	4.32431	-1.47669	0.0029
TOX3	TOX high mobility group box family member 3 [Source:NCBI gene;Acc:415724]	4.81614	1.73402	-1.47376	0.00125
PXK	PX domain containing serine/threonine kinase like [Source:HGNC Symbol;Acc:HGNC:23326]	145.783	52.6858	-1.46834	0.00005
IRF8	interferon regulatory factor 8 [Source:NCBI gene;Acc:396385]	224.971	81.3388	-1.46772	0.00005
RASL12	RAS like family 12 [Source:HGNC Symbol;Acc:HGNC:30289]	1.98311	0.717193	-1.46733	0.0449
SMCHD1	structural maintenance of chromosomes flexible hinge domain containing 1 [Source:HGNC Symbol;Acc:HGNC:29090]	310.369	112.596	-1.46283	0.0004
DNASE1L3	deoxyribonuclease 1 like 3 [Source:NCBI gene;Acc:416008]	22.3739	8.12317	-1.4617	0.00075
G0S2	G0/G1 switch 2 [Source:NCBI gene;Acc:419860]	5.27803	1.92026	-1.4587	0.036
CIDEA	cell death-inducing DFFA-like effector a [Source:NCBI gene;Acc:768659]	54.6249	19.877	-1.45846	0.00005
KAT2A	lysine acetyltransferase 2A [Source:HGNC Symbol;Acc:HGNC:4201]	31.6626	11.5384	-1.45634	0.00005
HELB	DNA helicase B [Source:HGNC Symbol;Acc:HGNC:17196]	49.5038	18.0506	-1.45549	0.00005
GREB1L	GREB1 like retinoic acid receptor coactivator [Source:HGNC Symbol;Acc:HGNC:31042]	12.1108	4.41968	-1.45428	0.00005
ENSGALG00000051546		22.5917	8.25798	-1.45193	0.00005
TCIM	transcriptional and immune response regulator [Source:HGNC Symbol;Acc:HGNC:1357]	378.591	138.822	-1.4474	0.00005
ACSL4	acyl-CoA synthetase long chain family member 4 [Source:HGNC Symbol;Acc:HGNC:3571]	106.339	39.0269	-1.44613	0.00015
ARHGAP8	Rho GTPase activating protein 8 [Source:NCBI gene;Acc:418239]	14.8801	5.46991	-1.4438	0.00025
KIAA1143	KIAA1143 [Source:NCBI gene;Acc:420709]	65.1009	23.9486	-1.44274	0.00005
RFX5	regulatory factor X5 [Source:HGNC Symbol;Acc:HGNC:9986]	6.44958	2.37298	-1.44251	0.032

ATP1B4	ATPase Na+/K+ transporting family member beta 4 [Source:NCBI gene;Acc:422365]	11.1434	4.10871	-1.43943	0.00065
ATP10D	ATPase phospholipid transporting 10D (putative) [Source:HGNC Symbol;Acc:HGNC:13549]	31.4833	11.6163	-1.43844	0.00005
AKAP6	A-kinase anchoring protein 6 [Source:HGNC Symbol;Acc:HGNC:376]	5.81692	2.14711	-1.43786	0.0008
ANKRD9	ankyrin repeat domain 9 [Source:HGNC Symbol;Acc:HGNC:20096]	16.4722	6.08339	-1.43709	0.00565
ENSGALG00000001894		10.0921	3.73045	-1.4358	0.0009
ENSGALG00000054657		18.3936	6.81562	-1.43229	0.00265
GSTA3	glutathione S-transferase alpha 3 [Source:NCBI gene;Acc:414896]	112.636	41.7411	-1.43213	0.00025
STAT4	signal transducer and activator of transcription 4 [Source:NCBI gene;Acc:768406]	23.8009	8.83291	-1.43006	0.00005
SPOCK3	SPARC (osteonectin), cwcv and kazal like domains proteoglycan 3 [Source:HGNC Symbol;Acc:HGNC:13565]	6.09398	2.26219	-1.42966	0.0072
ENSGALG00000050441	cytokine receptor common subunit beta-like [Source:NCBI gene;Acc:771315]	49.7223	18.4823	-1.42775	0.00045
FOSL2	FOS like 2, AP-1 transcription factor subunit [Source:NCBI gene;Acc:421416]	43.1779	16.0608	-1.42675	0.00005
ENSGALG00000048091	interferon-induced protein 35 [Source:NCBI gene;Acc:420011]	57.5547	21.4092	-1.4267	0.0017
GPR20	G protein-coupled receptor 20 [Source:HGNC Symbol;Acc:HGNC:4475]	3.94308	1.47195	-1.42159	0.02425
GFPT2	glutamine-fructose-6-phosphate transaminase 2 [Source:HGNC Symbol;Acc:HGNC:4242]	36.3229	13.5623	-1.42128	0.00005
ENSGALG00000011190	Placenta-specific gene 8-like 2 [Source:NCBI gene;Acc:100857411]	293.858	109.884	-1.41914	0.00005
GPR137B	G protein-coupled receptor 137B [Source:NCBI gene;Acc:421511]	69.2062	25.9232	-1.41666	0.0001
CTSS	cathepsin S [Source:NCBI gene;Acc:425657]	309.648	116.021	-1.41624	0.00005
LVRN	laeverin [Source:HGNC Symbol;Acc:HGNC:26904]	3.10372	1.16705	-1.41113	0.01695

IER5	immediate early response 5 [Source:HGNC Symbol;Acc:HGNC:5393]	64.623	24.3316	-1.40922	0.0004
SYTL5	synaptotagmin like 5 [Source:HGNC Symbol;Acc:HGNC:15589]	2.38842	0.90018	-1.40777	0.0216
PRELID3A	PRELI domain containing 3A [Source:NCBI gene;Acc:421035]	8.38121	3.16036	-1.40707	0.0031
VMP1	vacuole membrane protein 1 [Source:HGNC Symbol;Acc:HGNC:29559]	123.832	46.7395	-1.40567	0.00005
FOXS1	forkhead box S1 [Source:HGNC Symbol;Acc:HGNC:3735]	9.46713	3.57509	-1.40495	0.0297
ENSGALG00000049690		2.06158	0.778952	-1.40414	0.0408
TMPRSS9	transmembrane serine protease 9 [Source:HGNC Symbol;Acc:HGNC:30079]	1.84056	0.696365	-1.40223	0.0158
ENSGALG00000053296	chromosome 3 open reading frame, human C8orf80 [Source:NCBI gene;Acc:422000]	104.894	39.7102	-1.40135	0.00005
NCOA7	nuclear receptor coactivator 7 [Source:NCBI gene;Acc:421717]	58.9523	22.3239	-1.40096	0.00005
CD80	CD80 molecule [Source:NCBI gene;Acc:768950]	24.9839	9.48095	-1.3979	0.00275
ENSGALG00000039008	uncharacterized LOC776594 [Source:NCBI gene;Acc:776594]	154.336	58.7091	-1.39442	0.00005
BST1	bone marrow stromal cell antigen 1 [Source:NCBI gene;Acc:422828]	28.246	10.7705	-1.39096	0.0028
C1QA	complement C1q A chain [Source:HGNC Symbol;Acc:HGNC:1241]	105.035	40.0686	-1.39033	0.00005
CSF1	colony stimulating factor 1 [Source:NCBI gene;Acc:100499189]	10.608	4.04897	-1.38953	0.00085
AGTR1	angiotensin II receptor type 1 [Source:NCBI gene;Acc:396065]	5.00477	1.91135	-1.38871	0.0488
TNFRSF6B	TNF receptor superfamily member 6b [Source:HGNC Symbol;Acc:HGNC:11921]	5.90661	2.26077	-1.38552	0.006
CHRNB4	cholinergic receptor nicotinic beta 4 subunit [Source:NCBI gene;Acc:395613]	3.05544	1.17012	-1.38472	0.0356
PIM3	Pim-3 proto-oncogene, serine/threonine kinase [Source:HGNC Symbol;Acc:HGNC:19310]	36.3038	13.9142	-1.38356	0.00005
C1H21ORF91	chromosome 1 open reading frame, human C21orf91 [Source:NCBI gene;Acc:395489]	75.5195	28.9689	-1.38234	0.00005

ETV6	ETS variant 6 [Source:NCBI gene;Acc:395750]	75.1738	28.9197	-1.37818	0.0496
MOB3C	MOB kinase activator 3C [Source:HGNC Symbol;Acc:HGNC:29800]	21.959	8.45604	-1.37676	0.00015
F13A1	coagulation factor XIII A chain [Source:NCBI gene;Acc:395420]	106.073	40.863	-1.37619	0.0279
ENSGALG00000053107		4.39051	1.69158	-1.37602	0.00635
VLDLR	very low density lipoprotein receptor [Source:NCBI gene;Acc:396154]	86.2635	33.2401	-1.37583	0.00005
ENSGALG00000045940	nuclear GTPase, germinal center associated [Source:NCBI gene;Acc:422002]	25.3233	9.80491	-1.36889	0.0001
TMEM140	transmembrane protein 140 [Source:HGNC Symbol;Acc:HGNC:21870]	143.871	55.7144	-1.36865	0.00005
FOXO6	forkhead box O6 [Source:HGNC Symbol;Acc:HGNC:24814]	3.85283	1.49378	-1.36695	0.0047
ARHGEF28	Rho guanine nucleotide exchange factor 28 [Source:HGNC Symbol;Acc:HGNC:30322]	28.9894	11.2739	-1.36254	0.00005
SOX18	SRY-box 18 [Source:NCBI gene;Acc:374200]	132.581	51.6647	-1.35962	0.0001
C5H11ORF96	chromosome 5 open reading frame, human C11orf96 [Source:NCBI gene;Acc:770209]	186.978	72.8683	-1.35951	0.00005
CCR2	C-C motif chemokine receptor 2 [Source:NCBI gene;Acc:420696]	114.036	44.4672	-1.35868	0.00005
ENSGALG00000011450	leukotriene C4 synthase-like [Source:NCBI gene;Acc:770665]	5.39447	2.10468	-1.35788	0.02095
NLRC5	NLR family CARD domain containing 5 [Source:NCBI gene;Acc:100857413]	61.031	23.8243	-1.35711	0.00005
CLIP2	CAP-Gly domain containing linker protein 2 [Source:HGNC Symbol;Acc:HGNC:2586]	21.2554	8.31118	-1.3547	0.00005
F10	coagulation factor X [Source:NCBI gene;Acc:395876]	29.0478	11.3589	-1.35461	0.00015
NDC80	NDC80, kinetochore complex component [Source:NCBI gene;Acc:395134]	35.1558	13.7494	-1.35439	0.00005
RAPGEF5	Rap guanine nucleotide exchange factor 5 [Source:HGNC Symbol;Acc:HGNC:16862]	52.9906	20.8043	-1.34885	0.0001
FMR1	fragile X mental retardation 1 [Source:HGNC Symbol;Acc:HGNC:3775]	179.909	70.6876	-1.34774	0.00005

TRPM8	transient receptor potential cation channel subfamily M member 8 [Source:NCBI gene;Acc:424025]	4.89997	1.92613	-1.34707	0.006
ART1	ADP-ribosyltransferase 1 [Source:NCBI gene;Acc:429485]	38.6791	15.2074	-1.34678	0.0007
CREM	cAMP responsive element modulator [Source:NCBI gene;Acc:378903]	82.2757	32.3571	-1.34638	0.00005
SYNJ2BP	synaptojanin 2 binding protein [Source:NCBI gene;Acc:430724]	62.4005	24.6012	-1.34283	0.00005
ENSGALG00000044466	receptor-interacting serine-threonine kinase 3 [Source:NCBI gene;Acc:415708]	9.75856	3.86135	-1.33756	0.0035
ADAMTS8	ADAM metallopeptidase with thrombospondin type 1 motif 8 [Source:HGNC Symbol;Acc:HGNC:224]	27.7607	10.9986	-1.33572	0.00005
KALRN	kalirin RhoGEF kinase [Source:HGNC Symbol;Acc:HGNC:4814]	14.9686	5.93657	-1.33424	0.00005
CHRNA6	cholinergic receptor nicotinic alpha 6 subunit [Source:NCBI gene;Acc:396321]	17.5419	6.96522	-1.33256	0.0004
ADGRG5	adhesion G protein-coupled receptor G5 [Source:HGNC Symbol;Acc:HGNC:19010]	2.34356	0.931278	-1.33142	0.0212
C3AR1	complement C3a receptor 1 [Source:NCBI gene;Acc:418198]	54.644	21.8285	-1.32385	0.00015
ENSGALG00000007381		83.738	33.5718	-1.31863	0.00025
PRPS2	phosphoribosyl pyrophosphate synthetase 2 [Source:NCBI gene;Acc:418639]	3.749	1.5054	-1.31636	0.0231
IL2RA	interleukin 2 receptor subunit alpha [Source:NCBI gene;Acc:395294]	24.0064	9.64588	-1.31543	0.00105
IL18RAP	interleukin 18 receptor accessory protein [Source:HGNC Symbol;Acc:HGNC:5989]	4.40565	1.77184	-1.31411	0.02105
ENSGALG00000020986	G-protein coupled receptor 35-like [Source:NCBI gene;Acc:112532977]	3.53362	1.42154	-1.31369	0.0453
FGB	fibrinogen beta chain [Source:NCBI gene;Acc:373926]	7.21036	2.90155	-1.31325	0.0048
TLR21	Toll-like receptor 21 [Source:NCBI gene;Acc:415623]	13.2279	5.32396	-1.31301	0.00025
IL10RB	interleukin 10 receptor subunit beta [Source:NCBI gene;Acc:395663]	53.4834	21.6098	-1.30741	0.00005
TMEM123	transmembrane protein 123 [Source:NCBI gene;Acc:418984]	154.831	62.7542	-1.30291	0.0001

ENSGALG00000050631		50.741	20.6167	-1.29934	0.00005
FAM149A	family with sequence similarity 149 member A [Source:HGNC Symbol;Acc:HGNC:24527]	24.7245	10.0554	-1.29797	0.00015
TOR1AIP2	torsin 1A interacting protein 2 [Source:HGNC Symbol;Acc:HGNC:24055]	19.2722	7.86087	-1.29376	0.00005
CCDC85A	coiled-coil domain containing 85A [Source:HGNC Symbol;Acc:HGNC:29400]	22.9085	9.36208	-1.29098	0.00015
HACD4	3-hydroxyacyl-CoA dehydratase 4 [Source:HGNC Symbol;Acc:HGNC:20920]	28.9615	11.8698	-1.28684	0.0012
BCAT1	branched chain amino acid transaminase 1 [Source:HGNC Symbol;Acc:HGNC:976]	20.3692	8.35155	-1.28627	0.0026
STARD3NL	STARD3 N-terminal like [Source:HGNC Symbol;Acc:HGNC:19169]	79.2765	32.5238	-1.2854	0.00015
CATHL1	cathelicidin-1 [Source:NCBI gene;Acc:414337]	13.7085	5.62612	-1.28486	0.02055
CARS	cysteinyl-tRNA synthetase [Source:NCBI gene;Acc:423086]	79.2099	32.5225	-1.28424	0.0001
C15orf48	chromosome 15 open reading frame 48 [Source:HGNC Symbol;Acc:HGNC:29898]	159.084	65.445	-1.28143	0.0001
TASOR2	transcription activation suppressor family member 2 [Source:HGNC Symbol;Acc:HGNC:23484]	23.732	9.76887	-1.28057	0.03285
TFCP2L1	transcription factor CP2 like 1 [Source:HGNC Symbol;Acc:HGNC:17925]	3.95244	1.62769	-1.27992	0.0287
ENSGALG00000005964	family with sequence similarity 162, member B [Source:NCBI gene;Acc:422224]	42.259	17.4098	-1.27936	0.0003
ENSGALG00000026092	histamine H3 receptor-like [Source:NCBI gene;Acc:428525]	11.9634	4.93406	-1.27778	0.0062
LITAF	lipopolysaccharide induced TNF factor [Source:NCBI gene;Acc:374125]	36.0907	14.8858	-1.27769	0.00045
B3GNT4	UDP-GlcNAc:betaGal beta-1,3-N-acetylglucosaminyltransferase 4 [Source:HGNC Symbol;Acc:HGNC:15683]	19.4957	8.05818	-1.27463	0.00065
FANCB	Fanconi anemia complementation group B [Source:NCBI gene;Acc:418628]	3.42297	1.41568	-1.27375	0.0301
PSTPIP1	proline-serine-threonine phosphatase interacting protein 1	27.8758	11.5355	-1.27293	0.0001

	[Source:HGNC Symbol;Acc:HGNC:9580]				
TXNDC16	thioredoxin domain containing 16 [Source:HGNC Symbol;Acc:HGNC:19965]	17.7528	7.36248	-1.26978	0.0002
UCK2	uridine-cytidine kinase 2 [Source:NCBI gene;Acc:424406]	11.1111	4.61182	-1.26859	0.004
ICA1	islet cell autoantigen 1 [Source:HGNC Symbol;Acc:HGNC:5343]	19.508	8.09867	-1.26831	0.00055
CD86	CD86 molecule [Source:NCBI gene;Acc:427944]	5.83569	2.42713	-1.26565	0.01845
CD164L2	CD164 molecule like 2 [Source:HGNC Symbol;Acc:HGNC:32043]	5.69635	2.36991	-1.26521	0.0187
PRMT8	protein arginine methyltransferase 8 [Source:HGNC Symbol;Acc:HGNC:5188]	11.7099	4.89538	-1.25824	0.00355
TCIRG1	T-cell immune regulator 1, ATPase H+ transporting V0 subunit a3 [Source:NCBI gene;Acc:395472]	33.2296	13.9212	-1.25519	0.00005
ENSGALG00000047665	extensin-like [Source:NCBI gene;Acc:112533549]	1.78784	0.750236	-1.2528	0.00725
ENSGALG00000053867		46.5882	19.5894	-1.24989	0.00005
BIRC3	baculoviral IAP repeat containing 3 [Source:NCBI gene;Acc:374012]	613.27	258.082	-1.24869	0.0034
MMD	monocyte to macrophage differentiation associated [Source:NCBI gene;Acc:417397]	135.347	56.9712	-1.24836	0.00005
AOAH	acyloxyacyl hydrolase [Source:HGNC Symbol;Acc:HGNC:548]	5.21704	2.20152	-1.24473	0.0078
ACKR2	atypical chemokine receptor 2 [Source:HGNC Symbol;Acc:HGNC:1565]	5.32334	2.24752	-1.244	0.0264
ADGRF2	adhesion G protein-coupled receptor F2 [Source:HGNC Symbol;Acc:HGNC:18991]	2.70301	1.14186	-1.24318	0.04215
DALRD3	DALR anticodon binding domain containing 3 [Source:HGNC Symbol;Acc:HGNC:25536]	27.6764	11.7059	-1.24142	0.0003
ASL2	argininosuccinate lyase 2 [Source:NCBI gene;Acc:417545]	35.4185	14.988	-1.2407	0.01105
ENSGALG00000038923	fatty acyl-CoA hydrolase precursor, medium chain-like [Source:NCBI gene;Acc:415787]	11.637	4.92445	-1.24068	0.0111

PPARG	peroxisome proliferator-activated receptor gamma [Source:NCBI gene;Acc:373928]	24.7071	10.471	-1.23853	0.00165
ENSGALG00000003572	Rho/Rac guanine nucleotide exchange factor 18 [Source:NCBI gene;Acc:420132]	54.3233	23.0356	-1.23771	0.0002
ADGRF5	adhesion G protein-coupled receptor F5 [Source:HGNC Symbol;Acc:HGNC:19030]	80.3895	34.1734	-1.23413	0.00005
BCAP29	B-cell receptor associated protein 29 [Source:NCBI gene;Acc:417702]	185.857	79.0984	-1.23247	0.00005
DAPK2	death associated protein kinase 2 [Source:HGNC Symbol;Acc:HGNC:2675]	11.9832	5.11305	-1.22876	0.01615
MCOLN2	mucolipin 2 [Source:HGNC Symbol;Acc:HGNC:13357]	13.4157	5.72467	-1.22866	0.0018
IL20RA	interleukin 20 receptor subunit alpha [Source:HGNC Symbol;Acc:HGNC:6003]	17.8155	7.60542	-1.22803	0.0006
PLA2G6	phospholipase A2 group VI [Source:NCBI gene;Acc:418028]	17.9305	7.66821	-1.22545	0.00035
ARL4A	ADP ribosylation factor like GTPase 4A [Source:HGNC Symbol;Acc:HGNC:695]	158.563	67.8253	-1.22516	0.0003
CRTAM	cytotoxic and regulatory T-cell molecule [Source:NCBI gene;Acc:395342]	6.71131	2.87423	-1.22342	0.00495
TNFSF13B	tumor necrosis factor superfamily member 13b [Source:NCBI gene;Acc:374229]	17.241	7.38854	-1.22248	0.0004
ENSGALG00000006756	lipopolysaccharide binding protein [Source:NCBI gene;Acc:419293]	92.5964	39.6837	-1.22241	0.00665
KLF6	Kruppel like factor 6 [Source:NCBI gene;Acc:420463]	147.953	63.5346	-1.21952	0.0003
ENSGALG00000036286	HHIP like 1 [Source:NCBI gene;Acc:423448]	9.84486	4.23306	-1.21767	0.00135
ENSGALG00000032836	monoamine oxidase B [Source:NCBI gene;Acc:418561]	12.5656	5.40567	-1.21693	0.00445
IGSF1	immunoglobulin superfamily member 1 [Source:NCBI gene;Acc:419114]	12.5933	5.41943	-1.21644	0.00155
DGKQ	diacylglycerol kinase theta [Source:HGNC Symbol;Acc:HGNC:2856]	11.7244	5.05259	-1.21442	0.00135
IKBKE	inhibitor of nuclear factor kappa B kinase subunit epsilon [Source:HGNC Symbol;Acc:HGNC:14552]	8.79726	3.79276	-1.21381	0.0004

FN3K	fructosamine 3 kinase [Source:HGNC Symbol;Acc:HGNC:24822]	12.2623	5.29312	-1.21204	0.00235
CMTM6	CKLF like MARVEL transmembrane domain containing 6 [Source:HGNC Symbol;Acc:HGNC:19177]	98.9215	42.7384	-1.21075	0.0005
CSGALNACT2	chondroitin sulfate N-acetylgalactosaminyltransferase 2 [Source:HGNC Symbol;Acc:HGNC:24292]	47.4671	20.5437	-1.20823	0.00005
SIN3A	SIN3 transcription regulator family member A [Source:NCBI gene;Acc:415307]	40.0566	17.3663	-1.20575	0.00005
CREB5	cAMP responsive element binding protein 5 [Source:HGNC Symbol;Acc:HGNC:16844]	5.54517	2.40822	-1.20326	0.00665
TLR1B	toll-like receptor 1 family member B [Source:NCBI gene;Acc:771173]	44.6285	19.3864	-1.20292	0.00015
ENSGALG00000053626	natural cytotoxicity triggering receptor 1-like [Source:NCBI gene;Acc:107050390]	2.82251	1.22666	-1.20224	0.04735
ADGRG1	adhesion G protein-coupled receptor G1 [Source:HGNC Symbol;Acc:HGNC:4512]	50.8217	22.0961	-1.20165	0.0001
KCNJ15	potassium voltage-gated channel subfamily J member 15 [Source:HGNC Symbol;Acc:HGNC:6261]	26.5159	11.5444	-1.19966	0.00025
ST6GAL1	ST6 beta-galactoside alpha-2,6-sialyltransferase 1 [Source:NCBI gene;Acc:396169]	127.635	55.5919	-1.19908	0.00045
USP16	ubiquitin specific peptidase 16 [Source:HGNC Symbol;Acc:HGNC:12614]	58.2994	25.3951	-1.19893	0.00005
PHF2	PHD finger protein 2 [Source:HGNC Symbol;Acc:HGNC:8920]	26.6151	11.5936	-1.19892	0.0001
EIF2AK1	eukaryotic translation initiation factor 2 alpha kinase 1 [Source:NCBI gene;Acc:395360]	43.6168	19.0177	-1.19754	0.00015
SARAF	store-operated calcium entry associated regulatory factor [Source:NCBI gene;Acc:422624]	174.654	76.1549	-1.19749	0.00015
ACVR2A	activin A receptor type 2A [Source:NCBI gene;Acc:396324]	71.2818	31.0948	-1.19686	0.00005
ENSGALG00000000720	guanylate binding protein 1 [Source:NCBI gene;Acc:419563]	147.428	64.3311	-1.19642	0.00075
ATP10B	ATPase phospholipid transporting 10B (putative)	51.7705	22.6014	-1.19572	0.00005

	[Source:HGNC Symbol;Acc:HGNC:13543]				
PCSK2	proprotein convertase subtilisin/kexin type 2 [Source:HGNC Symbol;Acc:HGNC:8744]	15.3651	6.72015	-1.19309	0.00095
ENSGALG00000010718		16.9858	7.43564	-1.1918	0.00035
DRAM1	DNA damage regulated autophagy modulator 1 [Source:HGNC Symbol;Acc:HGNC:25645]	137.296	60.1121	-1.19156	0.00005
TAPBPL	TAP binding protein like [Source:NCBI gene;Acc:426340]	19.3809	8.48716	-1.19128	0.0007
ENSGALG00000050752		46.6831	20.4491	-1.19086	0.0395
ENSGALG00000006325	netrin-4-like [Source:NCBI gene;Acc:416036]	8.94616	3.92588	-1.18825	0.00435
CPT1A	carnitine palmitoyltransferase 1A [Source:NCBI gene;Acc:423118]	105.763	46.4535	-1.18698	0.00015
PLOD1	procollagen-lysine, 2-oxoglutarate 5-dioxygenase 1 [Source:NCBI gene;Acc:419485]	84.5828	37.1804	-1.18582	0.00125
GAB2	GRB2 associated binding protein 2 [Source:HGNC Symbol;Acc:HGNC:14458]	19.2245	8.45596	-1.18491	0.0002
MAP3K8	mitogen-activated protein kinase kinase kinase 8 [Source:HGNC Symbol;Acc:HGNC:6860]	15.8234	6.96506	-1.18385	0.0017
ENSGALG00000004814	rhophilin Rho GTPase binding protein 2 [Source:NCBI gene;Acc:415771]	45.7254	20.1306	-1.18361	0.0004
TLR4	toll like receptor 4 [Source:NCBI gene;Acc:417241]	27.3957	12.0619	-1.18349	0.0005
PDE4B	phosphodiesterase 4B [Source:NCBI gene;Acc:424700]	29.716	13.0926	-1.18249	0.00015
BEAN1	brain expressed, associated with NEDD4, 1 [Source:NCBI gene;Acc:415796]	7.35165	3.24246	-1.18098	0.0041
GNS	glucosamine (N-acetyl)-6-sulfatase [Source:NCBI gene;Acc:417829]	398.497	176.129	-1.17794	0.0018
RGS2	regulator of G-protein signaling 2 [Source:NCBI gene;Acc:378912]	19.9611	8.82406	-1.17768	0.00205
SHISA5	shisa family member 5 [Source:HGNC Symbol;Acc:HGNC:30376]	509.314	225.576	-1.17494	0.00005
TNFAIP6	TNF alpha induced protein 6 [Source:NCBI gene;Acc:424315]	8.56203	3.79422	-1.17415	0.01355
EVA1C	eva-1 homolog C [Source:HGNC Symbol;Acc:HGNC:13239]	38.1636	16.9161	-1.1738	0.00035

ENSGALG00000010979	hydroxysteroid 17-beta dehydrogenase 11 [Source:NCBI gene;Acc:422588]	19.785	8.77134	-1.17354	0.00345
HES6	hes family bHLH transcription factor 6 [Source:HGNC Symbol;Acc:HGNC:18254]	14.771	6.5494	-1.17333	0.01965
ITPKA	inositol-trisphosphate 3-kinase A [Source:NCBI gene;Acc:395694]	100.727	44.7203	-1.17145	0.0004
ENSGALG00000027822	uncharacterized LOC101747660 [Source:NCBI gene;Acc:101747660]	3.75232	1.66813	-1.16955	0.02415
ENSGALG00000042388		4.4253	1.97395	-1.16469	0.01845
TNFAIP3	TNF alpha induced protein 3 [Source:HGNC Symbol;Acc:HGNC:11896]	32.9156	14.6982	-1.16313	0.00015
ENSGALG00000000818	leucine rich repeat containing 32 [Source:NCBI gene;Acc:428121]	9.16246	4.09381	-1.16229	0.01585
TFEC	transcription factor EC [Source:NCBI gene;Acc:417773]	14.6188	6.5328	-1.16205	0.0062
CYP46A1	cytochrome P450 family 46 subfamily A member 1 [Source:NCBI gene;Acc:423450]	19.2937	8.63481	-1.15989	0.00065
PNPT1	polyribonucleotide nucleotidyltransferase 1 [Source:HGNC Symbol;Acc:HGNC:23166]	27.6974	12.4124	-1.15797	0.00005
ENSGALG00000001525	calcium binding and coiled-coil domain 2 [Source:NCBI gene;Acc:419993]	277.794	124.746	-1.15502	0.00075
RARRES2	retinoic acid receptor responder (tazarotene induced) 2 [Source:NCBI gene;Acc:420366]	133.659	60.0756	-1.15371	0.0044
PDGFD	platelet derived growth factor D [Source:NCBI gene;Acc:418978]	122.161	54.9218	-1.15333	0.00005
DUSP5	dual specificity phosphatase 5 [Source:HGNC Symbol;Acc:HGNC:3071]	34.4499	15.5006	-1.15218	0.00035
DLL4	delta like canonical Notch ligand 4 [Source:HGNC Symbol;Acc:HGNC:2910]	82.2781	37.0382	-1.15149	0.00015
B3GAT1L	beta-1,3-glucuronyltransferase 1-like [Source:NCBI gene;Acc:408180]	1.92688	0.868378	-1.14987	0.04685
PSTPIP2	proline-serine-threonine phosphatase interacting protein 2 [Source:HGNC Symbol;Acc:HGNC:9581]	17.8695	8.05655	-1.14927	0.0025
ENSGALG00000026776	TP53 induced glycolysis regulatory phosphatase [Source:NCBI gene;Acc:419040]	14.6891	6.62486	-1.14878	0.00955

DECR1	2,4-dienoyl-CoA reductase 1 [Source:HGNC Symbol;Acc:HGNC:2753]	84.5692	38.1562	-1.14821	0.00025
CDK5RAP1	CDK5 regulatory subunit associated protein 1 [Source:HGNC Symbol;Acc:HGNC:15880]	38.5862	17.4476	-1.14506	0.01505
ENSGALG00000039652	Rho guanine nucleotide exchange factor 37 [Source:NCBI gene;Acc:416149]	6.77878	3.06609	-1.14463	0.01935
NCF4	neutrophil cytosolic factor 4 [Source:NCBI gene;Acc:418052]	22.7686	10.3047	-1.14374	0.0017
LRP3	LDL receptor related protein 3 [Source:HGNC Symbol;Acc:HGNC:6695]	128.877	58.3429	-1.14337	0.00065
TGM2	transglutaminase 2 [Source:NCBI gene;Acc:396432]	251.752	114.004	-1.14292	0.0021
ORC1	origin recognition complex subunit 1 [Source:NCBI gene;Acc:424640]	9.4786	4.29389	-1.14239	0.003
PGAP1	post-GPI attachment to proteins 1 [Source:HGNC Symbol;Acc:HGNC:25712]	81.1616	36.8051	-1.14089	0.0003
FAM46A	family with sequence similarity 46 member A [Source:NCBI gene;Acc:421845]	222.719	101.03	-1.14044	0.00065
RASSF5	Ras association domain family member 5 [Source:NCBI gene;Acc:419844]	21.8402	9.91607	-1.13915	0.0028
PAPLN	papilin, proteoglycan like sulfated glycoprotein [Source:HGNC Symbol;Acc:HGNC:19262]	7.69631	3.49494	-1.1389	0.0029
ENSGALG00000052375		4.27169	1.94051	-1.13837	0.0182
DRP2	dystrophin related protein 2 [Source:HGNC Symbol;Acc:HGNC:3032]	4.20084	1.91403	-1.13406	0.00675
MAP3K6	mitogen-activated protein kinase kinase kinase 6 [Source:HGNC Symbol;Acc:HGNC:6858]	3.4516	1.57458	-1.1323	0.00535
CDHR1	cadherin related family member 1 [Source:NCBI gene;Acc:414847]	27.3491	12.4868	-1.13109	0.00225
RAB32	RAB32, member RAS oncogene family [Source:NCBI gene;Acc:421616]	35.829	16.3743	-1.12969	0.00055
CD164	CD164 molecule [Source:HGNC Symbol;Acc:HGNC:1632]	439.469	202.258	-1.11956	0.0022
ENSGALG00000047864		37.2418	17.1436	-1.11925	0.00485

CCDC82	coiled-coil domain containing 82 [Source:HGNC Symbol;Acc:HGNC:26282]	30.586	14.0878	-1.11843	0.0008
CEMIP	cell migration inducing hyaluronidase 1 [Source:HGNC Symbol;Acc:HGNC:29213]	10.0138	4.61396	-1.11791	0.0015 5
ENSGALG00000021 395	ATP-binding cassette, sub-family A (ABC1), member 9 [Source:NCBI gene;Acc:417443]	15.7392	7.26349	-1.11563	0.0039 5
PLA2R1	phospholipase A2 receptor 1 [Source:NCBI gene;Acc:404304]	66.6073	30.7433	-1.11541	0.0010 5
TRPV2	transient receptor potential cation channel subfamily V member 2 [Source:HGNC Symbol;Acc:HGNC:18082]	48.8046	22.5306	-1.11513	0.0006 5
NT5DC2	5'-nucleotidase domain containing 2 [Source:HGNC Symbol;Acc:HGNC:25717]	46.1379	21.3145	-1.11412	0.0001
PFKP	phosphofructokinase, platelet [Source:NCBI gene;Acc:428411]	126.609	58.8152	-1.10612	0.0003 5
ACE	angiotensin I converting enzyme [Source:NCBI gene;Acc:419953]	53.2407	24.7359	-1.10592	0.0003 5
TESK2	testis associated actin remodelling kinase 2 [Source:HGNC Symbol;Acc:HGNC:11732]	42.7525	19.8695	-1.10545	0.0001
ENSGALG00000013 625	interaction protein for cytohesin exchange factors 1 [Source:NCBI gene;Acc:421645]	22.3743	10.4083	-1.10411	0.0016
SLC22A3	solute carrier family 22 member 3 [Source:HGNC Symbol;Acc:HGNC:10967]	6.78393	3.16091	-1.10178	0.0266 5
SYP	synaptophysin [Source:NCBI gene;Acc:396359]	6.19078	2.88584	-1.10113	0.0241
CD38	CD38 molecule [Source:NCBI gene;Acc:422827]	15.2696	7.13177	-1.09833	0.0029 5
GDA	guanine deaminase [Source:HGNC Symbol;Acc:HGNC:4212]	19.7525	9.23172	-1.09736	0.0094 5
CAPN3	calpain 3 [Source:NCBI gene;Acc:423233]	4.11201	1.92238	-1.09695	0.019
TMEM255A	transmembrane protein 255A [Source:HGNC Symbol;Acc:HGNC:26086]	15.9371	7.45246	-1.0966	0.0043 5
SLA	Src like adaptor [Source:NCBI gene;Acc:378908]	29.4016	13.7544	-1.096	0.0014
LGALS3	lectin, galactoside-binding, soluble, 3 [Source:NCBI gene;Acc:373917]	56.9771	26.6611	-1.09565	0.0007
PLAU	plasminogen activator, urokinase [Source:NCBI gene;Acc:396424]	20.4023	9.56618	-1.09272	0.0013 5

MMR1L4	macrophage mannose receptor 1-like 4 [Source:NCBI gene;Acc:771888]	55.0749	25.852	-1.09112	0.00085
HYAL3	hyaluronidase 3 [Source:HGNC Symbol;Acc:HGNC:5322]	13.8503	6.50375	-1.09057	0.00075
OSBPL6	oxysterol binding protein like 6 [Source:HGNC Symbol;Acc:HGNC:16388]	13.0016	6.11084	-1.08925	0.0029
ENSGALG00000015183	Ras-related protein Rab-10-like [Source:NCBI gene;Acc:421099]	32.1011	15.1005	-1.08803	0.0079
ACKR4	atypical chemokine receptor 4 [Source:HGNC Symbol;Acc:HGNC:1611]	35.2636	16.6173	-1.08549	0.0023
IRF2	interferon regulatory factor 2 [Source:NCBI gene;Acc:396115]	157.448	74.2081	-1.08523	0.0002
ENSGALG00000045684	plasmalemma vesicle associated protein [Source:NCBI gene;Acc:100857417]	385.889	182.07	-1.08369	0.0017
ENSGALG00000032803	proto-oncogene Mas-like [Source:NCBI gene;Acc:776507]	16.7823	7.92223	-1.08296	0.00575
MST1	macrophage stimulating 1 (hepatocyte growth factor-like) [Source:NCBI gene;Acc:396135]	23.896	11.3024	-1.08014	0.0003
MTF2	metal response element binding transcription factor 2 [Source:NCBI gene;Acc:395174]	38.4059	18.1758	-1.07931	0.0003
RHOG	ras homolog family member G [Source:HGNC Symbol;Acc:HGNC:672]	10.7387	5.08787	-1.07769	0.00385
RPS6KL1	ribosomal protein S6 kinase like 1 [Source:HGNC Symbol;Acc:HGNC:20222]	5.98391	2.83533	-1.07757	0.0154
HSBP1L1	heat shock factor binding protein 1 like 1 [Source:NCBI gene;Acc:420812]	17.5227	8.30389	-1.07737	0.03915
CLSTN1	calsyntenin 1 [Source:NCBI gene;Acc:395372]	83.164	39.4596	-1.07558	0.00045
CH25H	cholesterol 25-hydroxylase [Source:NCBI gene;Acc:423788]	16.293	7.73444	-1.07488	0.01435
MGP	matrix Gla protein [Source:NCBI gene;Acc:395912]	2232.7	1059.94	-1.07481	0.00495
ARHGEF26	Rho guanine nucleotide exchange factor 26 [Source:HGNC Symbol;Acc:HGNC:24490]	29.6273	14.0694	-1.07437	0.0008
FAM13A	family with sequence similarity 13 member A [Source:HGNC Symbol;Acc:HGNC:19367]	44.4029	21.0949	-1.07376	0.00035
HS6ST1	heparan sulfate 6-O-sulfotransferase 1 [Source:NCBI gene;Acc:395141]	93.0246	44.2014	-1.07352	0.0004

WBP1L	WW domain binding protein 1 like [Source:HGNC Symbol;Acc:HGNC:23510]	58.5262	27.8156	-1.07319	0.0002
CASP18	initiator caspase [Source:NCBI gene;Acc:693266]	22.2592	10.5996	-1.07039	0.0007
MBNL3	muscleblind like splicing regulator 3 [Source:NCBI gene;Acc:422233]	129.674	61.8401	-1.06827	0.0031
NOS2	nitric oxide synthase 2 [Source:NCBI gene;Acc:395807]	35.8395	17.1128	-1.06647	0.0017
RFC1	replication factor C subunit 1 [Source:NCBI gene;Acc:422788]	59.937	28.6923	-1.06278	0.0003
TMEM35B	transmembrane protein 35B [Source:HGNC Symbol;Acc:HGNC:40021]	21.2267	10.1647	-1.06231	0.03675
ENSGALG00000003026		79.0735	37.8702	-1.06213	0.0014
RUBCNL	rubicon like autophagy enhancer [Source:HGNC Symbol;Acc:HGNC:20420]	31.6005	15.1382	-1.06175	0.00075
ADAR	adenosine deaminase RNA specific [Source:HGNC Symbol;Acc:HGNC:225]	71.9347	34.4872	-1.06063	0.00105
SERPINB1	serpin family B member 1 [Source:HGNC Symbol;Acc:HGNC:3311]	18.74	8.98728	-1.06016	0.0034
ABRACL	ABRA C-terminal like [Source:NCBI gene;Acc:770205]	76.9369	36.9015	-1.06	0.0045
HECW2	HECT, C2 and WW domain containing E3 ubiquitin protein ligase 2 [Source:HGNC Symbol;Acc:HGNC:29853]	74.8372	35.9509	-1.05773	0.00545
MTHFD2	methylenetetrahydrofolate dehydrogenase (NADP+ dependent) 2, methenyltetrahydrofolate cyclohydrolase [Source:NCBI gene;Acc:426126]	12.227	5.87706	-1.0569	0.0101
STAT3	signal transducer and activator of transcription 3 [Source:NCBI gene;Acc:420027]	137.32	66.0613	-1.05566	0.00085
NOD1	nucleotide binding oligomerization domain containing 1 [Source:NCBI gene;Acc:420677]	19.5452	9.40321	-1.05559	0.0003
UBA2	ubiquitin like modifier activating enzyme 2 [Source:NCBI gene;Acc:415784]	112.039	53.9413	-1.05454	0.0001
RNASET2	ribonuclease T2 [Source:NCBI gene;Acc:421569]	88.8147	42.7714	-1.05415	0.0006

KCNJ8	potassium voltage-gated channel subfamily J member 8 [Source:NCBI gene;Acc:395973]	16.5241	7.96058	-1.05363	0.0015
WDR37	WD repeat domain 37 [Source:HGNC Symbol;Acc:HGNC:31406]	65.6698	31.6594	-1.0526	0.0006
IRF1	interferon regulatory factor 1 [Source:NCBI gene;Acc:396384]	276.435	133.353	-1.05169	0.0026
DUS2	dihydrouridine synthase 2 [Source:HGNC Symbol;Acc:HGNC:26014]	13.6685	6.60127	-1.05004	0.02275
NACC2	NACC family member 2 [Source:NCBI gene;Acc:417128]	28.324	13.7061	-1.04721	0.0035
MCL1	BCL2 family apoptosis regulator [Source:NCBI gene;Acc:395674]	240.554	116.409	-1.04716	0.0017
SCNN1B	sodium channel epithelial 1 beta subunit [Source:HGNC Symbol;Acc:HGNC:10600]	13.3191	6.44653	-1.0469	0.0012
ENSGALG00000010554	regulator of microtubule dynamics 2 [Source:NCBI gene;Acc:421465]	9.45604	4.57898	-1.04621	0.0166
UVRAG	UV radiation resistance associated [Source:NCBI gene;Acc:419091]	65.9575	31.9687	-1.04488	0.0002
DDX27	DEAD-box helicase 27 [Source:NCBI gene;Acc:419308]	41.883	20.329	-1.04283	0.0004
PTAFR	platelet activating factor receptor [Source:HGNC Symbol;Acc:HGNC:9582]	36.8918	17.9109	-1.04246	0.0015
CSTF3	cleavage stimulation factor subunit 3 [Source:NCBI gene;Acc:421595]	37.9249	18.4534	-1.03926	0.0044
NOCT	nocturnin [Source:NCBI gene;Acc:404779]	26.2376	12.7673	-1.03918	0.00215
ANO8	anoctamin 8 [Source:HGNC Symbol;Acc:HGNC:29329]	6.34536	3.08793	-1.03906	0.00745
DOCK9	dedicator of cytokinesis 9 [Source:HGNC Symbol;Acc:HGNC:14132]	187.653	91.329	-1.03892	0.0065
TNFRSF1B	TNF receptor superfamily member 1B [Source:NCBI gene;Acc:395083]	44.1103	21.4697	-1.03881	0.00065
RPS6KA2	ribosomal protein S6 kinase A2 [Source:HGNC Symbol;Acc:HGNC:10431]	7.29455	3.55749	-1.03596	0.01755
SLC38A2	solute carrier family 38 member 2 [Source:NCBI gene;Acc:417807]	181.914	88.7234	-1.03587	0.0022
ANGPT1L	angiopoietin-related protein 1-like [Source:NCBI gene;Acc:395773]	23.2521	11.351	-1.03454	0.0097

LY6E	lymphocyte antigen 6 family member E [Source:NCBI gene;Acc:395550]	4230.39	2065.71	-1.03415	0.03755
LRRC1	leucine rich repeat containing 1 [Source:HGNC Symbol;Acc:HGNC:14307]	12.7516	6.22967	-1.03345	0.0046
ENSGALG00000037611	myosin IIIA-like [Source:NCBI gene;Acc:427419]	17.3144	8.47918	-1.02998	0.0026
IL17RA	interleukin 17 receptor A [Source:NCBI gene;Acc:418158]	50.417	24.7278	-1.02778	0.0008
PIK3CB	phosphatidylinositol-4,5-bisphosphate 3-kinase catalytic subunit beta [Source:NCBI gene;Acc:424826]	44.2605	21.7314	-1.02624	0.00035
TSPAN13	tetraspanin 13 [Source:NCBI gene;Acc:420595]	119.205	58.5559	-1.02556	0.00115
TSC22D4	TSC22 domain family member 4 [Source:NCBI gene;Acc:768091]	117.22	57.5908	-1.02531	0.0013
ATP10A	ATPase phospholipid transporting 10A (putative) [Source:HGNC Symbol;Acc:HGNC:13542]	33.6863	16.5672	-1.02383	0.0012
ENSGALG00000002099	6-phosphofructo-2-kinase/fructose-2,6-biphosphatase 4-like [Source:NCBI gene;Acc:100859653]	38.7863	19.0811	-1.0234	0.00175
SERPINB10	serpin family B member 10 [Source:HGNC Symbol;Acc:HGNC:8942]	10.4283	5.13215	-1.02287	0.0162
MMP9	matrix metallopeptidase 9 [Source:NCBI gene;Acc:395387]	8.54084	4.20622	-1.02185	0.0074
ARRDC1	arrestin domain containing 1 [Source:NCBI gene;Acc:427772]	31.6413	15.6079	-1.01953	0.0005
SEMA6C	semaphorin 6C [Source:HGNC Symbol;Acc:HGNC:10740]	7.62897	3.76793	-1.01772	0.0052
FKBP9	FK506 binding protein 9 [Source:NCBI gene;Acc:395652]	158.884	78.485	-1.01748	0.0029
TARP	TCR gamma alternate reading frame protein [Source:NCBI gene;Acc:776306]	45.7593	22.6144	-1.01682	0.00075
RNF216	ring finger protein 216 [Source:HGNC Symbol;Acc:HGNC:21698]	26.4337	13.0656	-1.0166	0.00045
SOUL	SOUL protein [Source:NCBI gene;Acc:395567]	23.84	11.8017	-1.01439	0.04325
EPB41L3	erythrocyte membrane protein band 4.1 like 3 [Source:HGNC Symbol;Acc:HGNC:3380]	22.3414	11.0694	-1.01314	0.0005
CD36	CD36 molecule [Source:NCBI gene;Acc:417730]	9.24904	4.58893	-1.01115	0.03595

EDA2R	ectodysplasin A2 receptor [Source:NCBI gene;Acc:422166]	4.4108	2.19214	-1.0087	0.04965
MGAT2	mannosyl (alpha-1,6-)-glycoprotein beta-1,2-N-acetylglucosaminyltransferase [Source:HGNC Symbol;Acc:HGNC:7045]	29.7279	14.7819	-1.00799	0.0061
TRAF3	TNF receptor associated factor 3 [Source:HGNC Symbol;Acc:HGNC:12033]	23.0926	11.4949	-1.00644	0.0055
LDLRAD4	low density lipoprotein receptor class A domain containing 4 [Source:NCBI gene;Acc:421044]	78.7349	39.202	-1.00608	0.00115
RFESD	Rieske Fe-S domain containing [Source:HGNC Symbol;Acc:HGNC:29587]	42.4977	85.0327	1.000633	0.00165
ENSGALG00000014664	KIAA0825 [Source:NCBI gene;Acc:427110]	6.55561	13.2045	1.010228	0.00495
TMEM267	transmembrane protein 267 [Source:HGNC Symbol;Acc:HGNC:26139]	7.75568	15.6269	1.010706	0.03085
BBOF1	basal body orientation factor 1 [Source:HGNC Symbol;Acc:HGNC:19855]	3.01355	6.07247	1.010819	0.0432
MEMO1	mediator of cell motility 1 [Source:NCBI gene;Acc:421483]	11.5809	23.4705	1.019101	0.04425
ENSGALG00000014722	C-type lectin-like receptor variant [Source:NCBI gene;Acc:769174]	7.46732	15.1365	1.019369	0.01465
STOML3	stomatin like 3 [Source:HGNC Symbol;Acc:HGNC:19420]	4.15733	8.43022	1.019913	0.02655
TEKT1	tektin 1 [Source:HGNC Symbol;Acc:HGNC:15534]	37.6692	76.6961	1.025768	0.00445
ENSGALG00000054531	proline rich 29 [Source:NCBI gene;Acc:419941]	3.36795	6.86889	1.028206	0.01145
AGR2	anterior gradient 2, protein disulphide isomerase family member [Source:HGNC Symbol;Acc:HGNC:328]	54.5085	111.234	1.029045	0.00175
ARMC4	armadillo repeat containing 4 [Source:HGNC Symbol;Acc:HGNC:25583]	9.26587	18.91	1.029151	0.0039
ENSGALG00000021855		5.81086	11.8619	1.029512	0.04365
SOX2	SRY-box 2 [Source:NCBI gene;Acc:396105]	6.22186	12.7109	1.030648	0.01465
ENSGALG00000027132		24.2484	49.5681	1.031522	0.0009
IQCD	IQ motif containing D [Source:HGNC Symbol;Acc:HGNC:25168]	1.97237	4.04049	1.0346	0.0335

ENSGALG00000005247	MAFF interacting protein (pseudogene) [Source:NCBI gene;Acc:416527]	4.29864	8.81368	1.035864	0.02005
CCDC167	coiled-coil domain containing 167 [Source:NCBI gene;Acc:421433]	6.85649	14.0833	1.038443	0.0155
SDK2	sidekick cell adhesion molecule 2 [Source:NCBI gene;Acc:395215]	1.8178	3.73924	1.040552	0.0051
SPAG6	sperm associated antigen 6 [Source:NCBI gene;Acc:420505]	7.50362	15.4628	1.043143	0.012
FZD10	frizzled class receptor 10 [Source:NCBI gene;Acc:373885]	2.62018	5.40705	1.045176	0.03455
EFHC1	EF-hand domain containing 1 [Source:HGNC Symbol;Acc:HGNC:16406]	4.59857	9.4933	1.045724	0.00795
INIP	INTS3 and NABP interacting protein [Source:NCBI gene;Acc:427331]	11.007	22.7346	1.046468	0.01075
CAMK4	calcium/calmodulin dependent protein kinase IV [Source:NCBI gene;Acc:723973]	2.67331	5.52492	1.047326	0.03715
IFT88	intraflagellar transport 88 [Source:HGNC Symbol;Acc:HGNC:20606]	5.26573	10.9138	1.051448	0.0062
PHOSPHO1	phosphoethanolamine/phosphocholine phosphatase [Source:NCBI gene;Acc:395650]	17.7557	36.8315	1.052658	0.0007
GABRA5	gamma-aminobutyric acid type A receptor alpha5 subunit [Source:HGNC Symbol;Acc:HGNC:4079]	4.91371	10.1937	1.052793	0.0143
FANCG	Fanconi anemia complementation group G [Source:NCBI gene;Acc:378893]	1.83552	3.81193	1.054333	0.03835
ENSGALG00000006048	family with sequence similarity 64 member A [Source:NCBI gene;Acc:417685]	16.3295	34.0609	1.060636	0.00965
SCN3B	sodium voltage-gated channel beta subunit 3 [Source:HGNC Symbol;Acc:HGNC:20665]	5.60886	11.7019	1.060963	0.0068
ARHGAP39	Rho GTPase activating protein 39 [Source:HGNC Symbol;Acc:HGNC:29351]	2.8452	5.93961	1.061838	0.00185
PRSS35	serine protease 35 [Source:HGNC Symbol;Acc:HGNC:21387]	6.59363	13.7729	1.062688	0.02
DMRT2	doublesex and mab-3 related transcription factor 2 [Source:HGNC Symbol;Acc:HGNC:2935]	3.3173	6.93331	1.063535	0.0188

SCD5	stearoyl-CoA desaturase 5 [Source:HGNC Symbol;Acc:HGNC:21088]	6.52753	13.6589	1.065232	0.00455
WNT1	Wnt family member 1 [Source:HGNC Symbol;Acc:HGNC:12774]	1.74955	3.66657	1.067447	0.0351
SLC43A3	solute carrier family 43 member 3 [Source:HGNC Symbol;Acc:HGNC:17466]	22.1552	46.4323	1.067483	0.00085
ENSGALG00000007833	tetratricopeptide repeat domain 12 [Source:NCBI gene;Acc:770784]	3.12273	6.54769	1.068178	0.01845
ENSGALG00000030853		2.62005	5.49698	1.069045	0.02155
PLPPR1	phospholipid phosphatase related 1 [Source:HGNC Symbol;Acc:HGNC:25993]	2.44223	5.13379	1.071825	0.02645
GFM2	G elongation factor mitochondrial 2 [Source:HGNC Symbol;Acc:HGNC:29682]	4.47771	9.42353	1.073506	0.0087
ENSGALG00000049191		4.05819	8.56624	1.077826	0.0416
MBLAC2	metallo-beta-lactamase domain containing 2 [Source:NCBI gene;Acc:431565]	11.2018	23.6943	1.080809	0.00055
ENSGALG00000016268	chromosome 1 open reading frame, human CXORF59 [Source:NCBI gene;Acc:418583]	3.2879	6.95675	1.081247	0.0034
BPGM	bisphosphoglycerate mutase [Source:NCBI gene;Acc:418172]	12.7233	26.9668	1.083711	0.0005
ENSGALG00000051734		4.07871	8.65042	1.084657	0.02465
GMPR2	guanosine monophosphate reductase 2 [Source:NCBI gene;Acc:420842]	6.52372	13.8438	1.085473	0.0029
ENSGALG00000044070	coiled-coil domain containing 78 [Source:NCBI gene;Acc:769634]	3.36695	7.14609	1.085712	0.0157
ENSGALG00000048396		5.46057	11.6004	1.087051	0.01835
ZBTB7C	zinc finger and BTB domain containing 7C [Source:HGNC Symbol;Acc:HGNC:31700]	3.35178	7.12414	1.087788	0.00675
IGFBP2	insulin like growth factor binding protein 2 [Source:NCBI gene;Acc:396315]	3.33676	7.09911	1.08919	0.01205
ADGRL3	adhesion G protein-coupled receptor L3 [Source:HGNC Symbol;Acc:HGNC:20974]	2.27515	4.84548	1.090678	0.0064
TFB2M	transcription factor B2, mitochondrial [Source:HGNC Symbol;Acc:HGNC:18559]	11.2616	23.9998	1.091611	0.0027

TNFAIP8L1	TNF alpha induced protein 8 like 1 [Source:NCBI gene;Acc:420162]	11.2686	24.1	1.096725	0.0018
IQCH	IQ motif containing H [Source:HGNC Symbol;Acc:HGNC:25721]	3.03726	6.50193	1.098098	0.01145
BCL11A	B-cell CLL/lymphoma 11A [Source:NCBI gene;Acc:421199]	3.42515	7.33399	1.098431	0.00115
DYDC2	DPY30 domain containing 2 [Source:NCBI gene;Acc:423627]	20.4075	43.7851	1.101341	0.0025
ENSGALG00000052894		4.21628	9.06062	1.103639	0.007
ENSGALG00000054096		14.6784	31.5976	1.10612	0.02655
SLC25A16	solute carrier family 25 member 16 [Source:HGNC Symbol;Acc:HGNC:10986]	61.083	131.52	1.106439	0.0005
CCDC171	coiled-coil domain containing 171 [Source:HGNC Symbol;Acc:HGNC:29828]	5.79115	12.5175	1.112025	0.00055
C14H16orf45	chromosome 14 C16orf45 homolog [Source:NCBI gene;Acc:416594]	3.42644	7.41336	1.113417	0.0369
DNAH5	dynein axonemal heavy chain 5 [Source:HGNC Symbol;Acc:HGNC:2950]	11.4035	24.7434	1.117567	0.00155
CFAP45	cilia and flagella associated protein 45 [Source:HGNC Symbol;Acc:HGNC:17229]	1.82459	3.9658	1.12004	0.04645
FOXJ1	forkhead box J1 [Source:NCBI gene;Acc:770009]	18.8754	41.0998	1.122624	0.0023
DHFR	dihydrofolate reductase [Source:NCBI gene;Acc:427317]	3.47075	7.56735	1.124541	0.0267
PACRG	parkin coregulated [Source:HGNC Symbol;Acc:HGNC:19152]	5.63128	12.3633	1.134529	0.01825
LRRC34	leucine rich repeat containing 34 [Source:HGNC Symbol;Acc:HGNC:28408]	6.2763	13.82	1.138771	0.00595
NREP	neuronal regeneration related protein [Source:NCBI gene;Acc:396353]	139.22	306.722	1.139565	0.00045
VWA3A	von Willebrand factor A domain containing 3A [Source:HGNC Symbol;Acc:HGNC:27088]	4.4674	9.87831	1.144829	0.00255
ZNF608	zinc finger protein 608 [Source:HGNC Symbol;Acc:HGNC:29238]	3.80258	8.41651	1.146244	0.00065
PURG	purine rich element binding protein G [Source:HGNC Symbol;Acc:HGNC:17930]	4.1816	9.26289	1.147407	0.01195

IGF-I	insulin like growth factor 1 [Source:NCBI gene;Acc:418090]	6.10158	13.5164	1.147456	0.0162
SLF1	SMC5-SMC6 complex localization factor 1 [Source:HGNC Symbol;Acc:HGNC:25408]	4.4478	9.89087	1.153006	0.0062
FAIM2	Fas apoptotic inhibitory molecule 2 [Source:HGNC Symbol;Acc:HGNC:17067]	1.54592	3.45477	1.160124	0.04035
SLC25A42	solute carrier family 25 member 42 [Source:HGNC Symbol;Acc:HGNC:28380]	6.76922	15.1456	1.161837	0.0008
ENSGALG00000054733		6.43067	14.3913	1.162156	0.0017
SNCAIP	synuclein alpha interacting protein [Source:HGNC Symbol;Acc:HGNC:11139]	3.10145	6.94991	1.164051	0.0036
LRRC43	leucine rich repeat containing 43 [Source:HGNC Symbol;Acc:HGNC:28562]	1.41464	3.17252	1.165194	0.03475
ENSGALG00000037464	WD repeat domain 27 [Source:NCBI gene;Acc:100857387]	4.76682	10.6908	1.165271	0.0043
ENSGALG00000050110		3.4283	7.70318	1.167961	0.0197
CD180	CD180 molecule [Source:HGNC Symbol;Acc:HGNC:6726]	1.88332	4.23225	1.168147	0.02
CARD10	caspase recruitment domain family member 10 [Source:HGNC Symbol;Acc:HGNC:16422]	0.64207	1.444	1.169268	0.04015
ZMYND10	zinc finger MYND-type containing 10 [Source:HGNC Symbol;Acc:HGNC:19412]	6.53363	14.731	1.172899	0.04735
RASGRF1	Ras protein specific guanine nucleotide releasing factor 1 [Source:HGNC Symbol;Acc:HGNC:9875]	0.951518	2.14719	1.174147	0.01915
ENSGALG00000031869	retinitis pigmentosa 1 (autosomal dominant) [Source:NCBI gene;Acc:421123]	14.2866	32.248	1.174547	0.00025
DTWD2	DTW domain containing 2 [Source:NCBI gene;Acc:427386]	0.891242	2.01311	1.175537	0.04225
EXOSC1	exosome component 1 [Source:HGNC Symbol;Acc:HGNC:17286]	6.28759	14.2066	1.175982	0.0458
CDKL1	cyclin dependent kinase like 1 [Source:HGNC Symbol;Acc:HGNC:1781]	3.12215	7.07151	1.179478	0.0192
ENSGALG00000015224	adenylate kinase 9 [Source:NCBI gene;Acc:771077]	1.45026	3.29151	1.182438	0.02915

ENSGALG00000049799		8.96441	20.3854	1.185256	0.0058
REEP6	receptor accessory protein 6 [Source:HGNC Symbol;Acc:HGNC:30078]	6.63141	15.1197	1.189042	0.04135
SUOX	sulfite oxidase [Source:HGNC Symbol;Acc:HGNC:11460]	22.5034	51.3789	1.191033	0.0005
NRXN3	neurexin 3 [Source:NCBI gene;Acc:423385]	0.939275	2.15214	1.196152	0.03505
CA13	carbonic anhydrase 13 [Source:HGNC Symbol;Acc:HGNC:14914]	176.114	403.547	1.196227	0.0006
ENSGALG00000029618		9.80568	22.5127	1.19905	0.03155
DRC1	dynein regulatory complex subunit 1 [Source:HGNC Symbol;Acc:HGNC:24245]	2.68204	6.1581	1.199155	0.00955
IQCA1	IQ motif containing with AAA domain 1 [Source:HGNC Symbol;Acc:HGNC:26195]	1.46988	3.37726	1.200155	0.0313
SALL1	spalt like transcription factor 1 [Source:NCBI gene;Acc:395446]	0.830889	1.91501	1.204624	0.027
RSPH1	radial spoke head component 1 [Source:HGNC Symbol;Acc:HGNC:12371]	6.82705	15.7508	1.206091	0.00455
DACT2	dishevelled binding antagonist of beta catenin 2 [Source:HGNC Symbol;Acc:HGNC:21231]	1.5796	3.64635	1.206894	0.0103
ATP8A2	ATPase phospholipid transporting 8A2 [Source:HGNC Symbol;Acc:HGNC:13533]	0.810477	1.87714	1.211693	0.0461
ENSGALG00000007007	kinase non-catalytic C-lobe domain containing 1 [Source:NCBI gene;Acc:423824]	2.12914	4.93708	1.213387	0.01115
ENSGALG00000043947	G-protein coupled receptor 183-like [Source:NCBI gene;Acc:431250]	1.55964	3.61786	1.213924	0.017
ENSGALG00000002108	envoplakin [Source:NCBI gene;Acc:427805]	1.40794	3.27504	1.217927	0.00125
SAXO2	stabilizer of axonemal microtubules 2 [Source:HGNC Symbol;Acc:HGNC:33727]	8.14962	18.9763	1.219394	0.0013
FAM219A	family with sequence similarity 219 member A [Source:HGNC Symbol;Acc:HGNC:19920]	9.77971	22.7834	1.220119	0.0304
DENND5B	DENN domain containing 5B [Source:HGNC Symbol;Acc:HGNC:28338]	1.4444	3.36536	1.220291	0.0047
NSUN7	NOP2/Sun RNA methyltransferase family member	5.08811	11.8741	1.222616	0.00505

	7 [Source:HGNC Symbol;Acc:HGNC:25857]				
SPAG17	sperm associated antigen 17 [Source:HGNC Symbol;Acc:HGNC:26620]	2.43878	5.69199	1.222774	0.00365
ENSGALG00000001244	TBC1 domain family member 24-like [Source:NCBI gene;Acc:769242]	5.4412	12.7158	1.224625	0.0022
ENSGALG00000047773	chromosome 4 C2orf81 homolog [Source:NCBI gene;Acc:107056276]	1.61282	3.76931	1.224715	0.0283
CHST2	carbohydrate sulfotransferase 2 [Source:HGNC Symbol;Acc:HGNC:1970]	18.0435	42.4346	1.233762	0.0008
SIX2	SIX homeobox 2 [Source:NCBI gene;Acc:724086]	4.54005	10.6844	1.234726	0.02075
TPPP3	tubulin polymerization promoting protein family member 3 [Source:HGNC Symbol;Acc:HGNC:24162]	10.2966	24.2948	1.23848	0.0049
RASAL1	RAS protein activator like 1 [Source:HGNC Symbol;Acc:HGNC:9873]	1.67767	3.96305	1.240152	0.0154
SVOPL	SVOP like [Source:HGNC Symbol;Acc:HGNC:27034]	3.78052	8.93526	1.240925	0.01255
DRC7	dynein regulatory complex subunit 7 [Source:HGNC Symbol;Acc:HGNC:25289]	3.06251	7.25943	1.245142	0.0016
RIPOR3	RIPOR family member 3 [Source:HGNC Symbol;Acc:HGNC:16168]	69.4862	164.791	1.245839	0.00025
ENSGALG00000044151	MORN repeat containing 5 [Source:NCBI gene;Acc:772256]	3.01934	7.16203	1.246135	0.0477
DNAJB13	DnaJ heat shock protein family (Hsp40) member B13 [Source:HGNC Symbol;Acc:HGNC:30718]	3.49687	8.2958	1.246317	0.01075
ENSGALG00000019932	uncharacterized LOC101748060 [Source:NCBI gene;Acc:101748060]	4.46461	10.594	1.246641	0.00035
RIBC2	RIB43A domain with coiled-coils 2 [Source:HGNC Symbol;Acc:HGNC:13241]	3.16351	7.51929	1.24907	0.01345
ENSGALG00000019240		3.21833	7.65068	1.249276	0.0382
ELL2	elongation factor for RNA polymerase II 2 [Source:HGNC Symbol;Acc:HGNC:17064]	1.92378	4.57417	1.249566	0.00915
ARHGAP40	Rho GTPase activating protein 40 [Source:NCBI gene;Acc:419168]	1.97685	4.70195	1.250056	0.02015

ENSGALG00000001963	serine/threonine/tyrosine interacting like 1 [Source:NCBI gene;Acc:417518]	3.60716	8.60161	1.253743	0.0249
SLC6A9	solute carrier family 6 member 9 [Source:NCBI gene;Acc:424576]	2.55186	6.09823	1.256841	0.0072
SLC46A3	solute carrier family 46 member 3 [Source:NCBI gene;Acc:418924]	10.6693	25.5489	1.259796	0.00025
FNDC1	fibronectin type III domain containing 1 [Source:HGNC Symbol;Acc:HGNC:21184]	6.28304	15.0821	1.263303	0.0006
SGCG	sarcoglycan gamma [Source:HGNC Symbol;Acc:HGNC:10809]	1.70604	4.09753	1.264103	0.04325
ENSGALG00000016646		2.01696	4.84452	1.264171	0.04455
GPR176	G protein-coupled receptor 176 [Source:HGNC Symbol;Acc:HGNC:32370]	3.91576	9.43562	1.268825	0.0118
PLEKHS1	pleckstrin homology domain containing S1 [Source:HGNC Symbol;Acc:HGNC:26285]	7.97503	19.2387	1.270449	0.00105
MYOM2	myomesin 2 [Source:NCBI gene;Acc:396034]	1.34151	3.2393	1.271824	0.0115
CYP1B1	cytochrome P450 family 1 subfamily B member 1 [Source:HGNC Symbol;Acc:HGNC:2597]	2.86145	6.91116	1.272181	0.0042
ST6GALNAC3	ST6 N-acetylgalactosaminide alpha-2,6-sialyltransferase 3 [Source:HGNC Symbol;Acc:HGNC:19343]	2.56017	6.21995	1.280663	0.01845
PRAG1	PEAK1 related, kinase-activating pseudokinase 1 [Source:HGNC Symbol;Acc:HGNC:25438]	1.17823	2.86609	1.282463	0.00665
RSPH6A	radial spoke head 6 homolog A [Source:HGNC Symbol;Acc:HGNC:14241]	3.02524	7.37208	1.285022	0.00485
ENSGALG00000011356	glutamate-rich protein 3 [Source:NCBI gene;Acc:424719]	14.6338	35.6909	1.286252	0.0003
ENSGALG00000020523	TOP1 binding arginine/serine rich protein [Source:NCBI gene;Acc:427342]	13.5777	33.2749	1.293195	0.00005
ENSGALG00000052859		8.18198	20.127	1.29861	0.00475
SUSD1	sushi domain containing 1 [Source:HGNC Symbol;Acc:HGNC:25413]	14.2008	35.0005	1.301403	0.00005
ADCY8	adenylate cyclase 8 [Source:HGNC Symbol;Acc:HGNC:239]	9.13833	22.5248	1.301512	0.0003

FRMD3	FERM domain containing 3 [Source:HGNC Symbol;Acc:HGNC:24125]	3.46234	8.54813	1.303861	0.00335
PPP1R3C	protein phosphatase 1 regulatory subunit 3C [Source:HGNC Symbol;Acc:HGNC:9293]	3.86506	9.55321	1.305495	0.00335
FEV	FEV transcription factor, ETS family member [Source:HGNC Symbol;Acc:HGNC:18562]	1.10476	2.73333	1.306927	0.02815
ENSGALG00000026294	phosphatidylinositol glycan anchor biosynthesis class Z [Source:NCBI gene;Acc:429144]	1.1807	2.92228	1.307452	0.04925
SULT1C3	sulfotransferase family 1C member 3 [Source:NCBI gene;Acc:395300]	4.6376	11.4936	1.30938	0.0104
KIF6	kinesin family member 6 [Source:HGNC Symbol;Acc:HGNC:21202]	1.94658	4.82711	1.310218	0.0361
SPDEF	SAM pointed domain containing ETS transcription factor [Source:HGNC Symbol;Acc:HGNC:17257]	3.60966	8.95947	1.31155	0.01395
ENSGALG00000045636	uncharacterized LOC427400 [Source:NCBI gene;Acc:427400]	0.669959	1.66328	1.311886	0.04255
DEUP1	deuterosome assembly protein 1 [Source:HGNC Symbol;Acc:HGNC:26344]	1.56387	3.89438	1.316273	0.0344
ZNF513	zinc finger protein 513 [Source:HGNC Symbol;Acc:HGNC:26498]	5.21789	13.0196	1.319147	0.01225
VIL1	villin 1 [Source:NCBI gene;Acc:396423]	0.994611	2.49524	1.326974	0.019
PERP1	PERP1, TP53 apoptosis effector [Source:NCBI gene;Acc:421683]	1.37577	3.46151	1.331162	0.0322
KCNT2	potassium sodium-activated channel subfamily T member 2 [Source:HGNC Symbol;Acc:HGNC:18866]	1.02277	2.58005	1.334917	0.0219
COL17A1	collagen type XVII alpha 1 chain [Source:NCBI gene;Acc:396503]	1.02782	2.59347	1.335296	0.0042
STRA6	stimulated by retinoic acid 6 [Source:NCBI gene;Acc:415301]	3.32858	8.4	1.335482	0.00095
PYCR1	pyrroline-5-carboxylate reductase 1 [Source:NCBI gene;Acc:769203]	3.97102	10.0394	1.338092	0.0013
ENSGALG00000046960		18.2011	46.1145	1.341195	0.03005
CPLANE1	ciliogenesis and planar polarity effector 1 [Source:HGNC Symbol;Acc:HGNC:25801]	6.26382	15.8767	1.341796	0.00005

MMP27	matrix metallopeptidase 27 [Source:NCBI gene;Acc:395850]	4.44034	11.2814	1.345204	0.00385
LPAR4	lysophosphatidic acid receptor 4 [Source:NCBI gene;Acc:422149]	3.62843	9.22941	1.346893	0.00305
CXCL14	C-X-C motif chemokine ligand 14 [Source:NCBI gene;Acc:395451]	3.40104	8.66555	1.349315	0.0158
SRCIN1	SRC kinase signaling inhibitor 1 [Source:HGNC Symbol;Acc:HGNC:29506]	1.14859	2.9376	1.354774	0.0037
GLIS1	GLIS family zinc finger 1 [Source:HGNC Symbol;Acc:HGNC:29525]	3.41247	8.78873	1.364838	0.00215
MGAT4D	MGAT4 family member D [Source:HGNC Symbol;Acc:HGNC:43619]	1.27188	3.2757	1.364841	0.03265
SELENOO	selenoprotein O [Source:NCBI gene;Acc:417745]	9.12652	23.6213	1.371952	0.00005
BARX2	BARX homeobox 2 [Source:NCBI gene;Acc:395714]	2.60032	6.77238	1.380974	0.03175
ENKUR	enkurin, TRPC channel interacting protein [Source:HGNC Symbol;Acc:HGNC:28388]	9.48654	24.7479	1.383352	0.00085
SPEF2	sperm flagellar 2 [Source:HGNC Symbol;Acc:HGNC:26293]	7.96446	20.8017	1.385053	0.0004
LRRC61	leucine rich repeat containing 61 [Source:HGNC Symbol;Acc:HGNC:21704]	1.4817	3.8723	1.385937	0.0432
RBM38	RNA binding motif protein 38 [Source:NCBI gene;Acc:768866]	23.5214	61.6456	1.390024	0.00005
ADAMTS19	ADAM metallopeptidase with thrombospondin type 1 motif 19 [Source:HGNC Symbol;Acc:HGNC:17111]	2.72664	7.15173	1.391168	0.00145
ENSGALG00000034761		2.16582	5.68884	1.393221	0.014
CCNA1	cyclin A1 [Source:HGNC Symbol;Acc:HGNC:1577]	1.70872	4.49275	1.394683	0.02615
CA3A	carbonic anhydrase 3A [Source:NCBI gene;Acc:420208]	2.2614	5.94803	1.395196	0.0441
DNM3	dynamin 3 [Source:HGNC Symbol;Acc:HGNC:29125]	1.24767	3.28397	1.396205	0.03605
RHAG	Rh associated glycoprotein [Source:NCBI gene;Acc:395118]	6.56817	17.3807	1.403923	0.0006
ENSGALG00000003176		3.2065	8.48874	1.404551	0.0225
ENSGALG00000001111		6.89635	18.2823	1.406543	0.00015

FRRS1	ferric chelate reductase 1 [Source:HGNC Symbol;Acc:HGNC:27622]	24.1148	64.3318	1.415613	0.00015
FIBIN	fin bud initiation factor homolog (zebrafish) [Source:NCBI gene;Acc:426933]	4.57652	12.2094	1.415669	0.00205
ENSGALG00000053276	coiled-coil domain containing 166 [Source:NCBI gene;Acc:426291]	0.840362	2.24364	1.416758	0.02485
ELAVL4	ELAV like RNA binding protein 4 [Source:NCBI gene;Acc:395634]	1.16103	3.11006	1.421537	0.01405
EEF1A2	eukaryotic translation elongation factor 1 alpha 2 [Source:NCBI gene;Acc:419244]	2.29592	6.16324	1.424617	0.00565
ANKEF1	ankyrin repeat and EF-hand domain containing 1 [Source:HGNC Symbol;Acc:HGNC:15803]	1.41055	3.79477	1.427755	0.0213
NHLH1	nescient helix-loop-helix 1 [Source:NCBI gene;Acc:373910]	0.666901	1.79859	1.431322	0.015
KRT13	keratin 13 [Source:NCBI gene;Acc:408040]	2.75258	7.43015	1.432607	0.00245
ENSGALG00000052336		5.80977	15.7256	1.436562	0.00035
E2F7	E2F transcription factor 7 [Source:HGNC Symbol;Acc:HGNC:23820]	1.37315	3.73699	1.444387	0.01255
HYDIN	HYDIN, axonemal central pair apparatus protein [Source:NCBI gene;Acc:427538]	2.61833	7.12654	1.444555	0.00005
CFAP77	cilia and flagella associated protein 77 [Source:HGNC Symbol;Acc:HGNC:33776]	2.31534	6.32055	1.448826	0.02045
CCNJ	cyclin J [Source:HGNC Symbol;Acc:HGNC:23434]	10.1241	27.6613	1.450075	0.00125
DNAI1	dynein axonemal intermediate chain 1 [Source:HGNC Symbol;Acc:HGNC:2954]	2.92579	8.00805	1.452625	0.00495
LIX1	limb and CNS expressed 1 [Source:NCBI gene;Acc:374264]	0.758742	2.0776	1.453237	0.04915
C9orf116	chromosome 9 open reading frame 116 [Source:HGNC Symbol;Acc:HGNC:28435]	8.43253	23.1992	1.460038	0.0032
CFAP65	cilia and flagella associated protein 65 [Source:HGNC Symbol;Acc:HGNC:25325]	4.09802	11.2932	1.462455	0.00005
ACTA1	actin, alpha 1, skeletal muscle [Source:NCBI gene;Acc:421534]	32.2716	89.0542	1.464419	0.00025

LNX1	ligand of numb-protein X 1 [Source:HGNC Symbol;Acc:HGNC:6657]	0.752709	2.08061	1.466842	0.0177
TP63	tumor protein p63 [Source:NCBI gene;Acc:374269]	2.22625	6.15849	1.467961	0.0017
UNC119	unc-119 lipid binding chaperone [Source:HGNC Symbol;Acc:HGNC:12565]	1.57462	4.35979	1.469255	0.02175
ENSGALG00000054650	coiled-coil domain-containing protein 180-like [Source:NCBI gene;Acc:112529956]	4.15302	11.597	1.481519	0.00205
AHR2	aryl hydrocarbon receptor 2 [Source:NCBI gene;Acc:424033]	0.592804	1.65606	1.482128	0.0364
PP2D1	protein phosphatase 2C like domain containing 1 [Source:HGNC Symbol;Acc:HGNC:28406]	2.01258	5.65036	1.489297	0.00635
ENSGALG00000049260	t-complex 10 [Source:NCBI gene;Acc:421567]	3.89474	11.018	1.500263	0.0001
SLC23A1	solute carrier family 23 member 1 [Source:HGNC Symbol;Acc:HGNC:10974]	1.50054	4.24882	1.50158	0.01545
BCHE	butyrylcholinesterase [Source:NCBI gene;Acc:395358]	3.06487	8.68838	1.503261	0.0005
ENSGALG00000044985	mitotic spindle positioning [Source:NCBI gene;Acc:420097]	11.808	33.5145	1.505021	0.00005
USH1C	USH1 protein network component harmonin [Source:HGNC Symbol;Acc:HGNC:12597]	1.26752	3.62392	1.515543	0.013
CHL1	cell adhesion molecule L1 like [Source:HGNC Symbol;Acc:HGNC:1939]	1.08579	3.10549	1.516076	0.00255
ENSGALG00000047341		1.26404	3.6463	1.528391	0.03485
SLC2A11	solute carrier family 2 member 11 [Source:NCBI gene;Acc:427704]	0.586053	1.69201	1.529635	0.0237
ENSGALG00000051776		2.20831	6.3783	1.530229	0.00005
FOLR1	folate receptor 1 [Source:NCBI gene;Acc:395638]	9.99733	28.9852	1.535702	0.00025
LDB3	LIM domain binding 3 [Source:NCBI gene;Acc:423610]	1.86331	5.4072	1.537014	0.00745
ENSGALG00000023824	lipase member M-like 5 [Source:NCBI gene;Acc:428958]	1.82728	5.31699	1.540912	0.0175
SNTN	sentan, cilia apical structure protein [Source:NCBI gene;Acc:416076]	9.03713	26.3163	1.54202	0.00025
ADRB1	adrenoceptor beta 1 [Source:HGNC Symbol;Acc:HGNC:285]	20.039	58.7568	1.551945	0.00005

NME1	NME/NM23 nucleoside diphosphate kinase 1 [Source:NCBI gene;Acc:422094]	0.756875	2.2257	1.556132	0.02295
FAT2	FAT atypical cadherin 2 [Source:HGNC Symbol;Acc:HGNC:3596]	1.01455	2.99995	1.564098	0.00005
ENSGALG00000051202	leucine-rich repeat-containing protein 51-like [Source:NCBI gene;Acc:107052453]	0.815574	2.42072	1.569549	0.0346
HTR1B	5-hydroxytryptamine receptor 1B [Source:NCBI gene;Acc:421858]	2.91888	8.7175	1.5785	0.00065
MYBPC1	myosin binding protein C, slow type [Source:HGNC Symbol;Acc:HGNC:7549]	1.21559	3.63278	1.579417	0.00325
FSIP1	fibrous sheath interacting protein 1 [Source:HGNC Symbol;Acc:HGNC:21674]	1.38506	4.15488	1.584858	0.0119
USP2	ubiquitin specific peptidase 2 [Source:HGNC Symbol;Acc:HGNC:12618]	0.598547	1.81609	1.601299	0.0139
ENSGALG00000021238	cytochrome P450 family 2 subfamily W member 1 [Source:NCBI gene;Acc:416453]	1.06476	3.2414	1.606089	0.0112
PCYT1B	phosphate cytidylyltransferase 1, choline, beta [Source:HGNC Symbol;Acc:HGNC:8755]	0.702117	2.14862	1.613627	0.039
SOX6	SRY-box 6 [Source:NCBI gene;Acc:423068]	30.3741	93.1329	1.616449	0.00005
GABRP	gamma-aminobutyric acid type A receptor pi subunit [Source:HGNC Symbol;Acc:HGNC:4089]	3.80226	11.7719	1.630418	0.0006
DCX	doublecortin [Source:NCBI gene;Acc:374242]	3.05296	9.47516	1.633942	0.00065
ENSGALG00000050176		0.629176	1.96091	1.639988	0.0396
SPACA9	sperm acrosome associated 9 [Source:NCBI gene;Acc:772069]	5.15613	16.2003	1.65166	0.0084
ENSGALG00000035854		1.10272	3.50026	1.666396	0.00945
BCAS1	breast carcinoma amplified sequence 1 [Source:HGNC Symbol;Acc:HGNC:974]	1.2408	3.94964	1.670451	0.0025
ENSGALG00000040415	actin, beta-like 2 [Source:NCBI gene;Acc:426652]	0.895011	2.85035	1.671162	0.0408
SPON2	spondin 2 [Source:HGNC Symbol;Acc:HGNC:11253]	24.3221	77.4938	1.671813	0.00005
PPIL6	peptidylprolyl isomerase like 6 [Source:HGNC Symbol;Acc:HGNC:21557]	3.81166	12.2035	1.678804	0.00495

TTC29	tetratricopeptide repeat domain 29 [Source:HGNC Symbol;Acc:HGNC:29936]	0.859126	2.77956	1.693915	0.03885
SLC38A11	solute carrier family 38 member 11 [Source:HGNC Symbol;Acc:HGNC:26836]	1.7378	5.71832	1.718329	0.0072
THRSP	thyroid hormone responsive [Source:NCBI gene;Acc:404755]	0.938424	3.12994	1.737823	0.03135
VEPH1	ventricular zone expressed PH domain containing 1 [Source:HGNC Symbol;Acc:HGNC:25735]	0.82574	2.76178	1.741839	0.03875
ENSGALG00000051229		1.93692	6.52879	1.753051	0.00365
ENSGALG00000052028		4.8967	16.5118	1.753616	0.0034
KIF24	kinesin family member 24 [Source:HGNC Symbol;Acc:HGNC:19916]	1.48074	5.03886	1.766779	0.0002
ANK1	ankyrin 1 [Source:HGNC Symbol;Acc:HGNC:492]	11.1529	38.0301	1.769723	0.00005
ENSGALG00000053886		21.5288	73.7028	1.775452	0.00005
ALDH1A1	aldehyde dehydrogenase 1 family member A1 [Source:NCBI gene;Acc:395264]	12.4434	42.83	1.783241	0.00005
TFAP2A	transcription factor AP-2 alpha [Source:NCBI gene;Acc:395982]	0.570254	1.97154	1.789646	0.0176
NOX1	NADPH oxidase 1 [Source:NCBI gene;Acc:422257]	0.637306	2.22648	1.804707	0.0178
CA3B	carbonic anhydrase 3B [Source:NCBI gene;Acc:420209]	21.6226	75.9012	1.811583	0.00005
VIPR2	vasoactive intestinal peptide receptor 2 [Source:NCBI gene;Acc:420453]	0.57268	2.02144	1.819582	0.0385
ENSGALG00000003466	aldo-keto reductase family 1, member B1-like [Source:NCBI gene;Acc:425137]	14.1473	50.0375	1.822483	0.0008
ENSGALG00000010457		2.01003	7.13488	1.827672	0.0188
APOBEC2	apolipoprotein B mRNA editing enzyme catalytic subunit 2 [Source:HGNC Symbol;Acc:HGNC:605]	0.851735	3.0281	1.829936	0.00915
KIRREL3	kirre like nephrin family adhesion molecule 3 [Source:HGNC Symbol;Acc:HGNC:23204]	0.405089	1.45925	1.848916	0.00505
ENSGALG00000054032		15.4351	56.2303	1.865133	0.02135
TNNC2	troponin C2, fast skeletal type [Source:NCBI gene;Acc:396434]	1.21473	4.44328	1.870989	0.0233

CRYBB3	crystallin beta B3 [Source:NCBI gene;Acc:396108]	1.33449	4.90905	1.879155	0.02
KRT14	keratin 14 [Source:NCBI gene;Acc:408039]	3.06953	11.2973	1.879888	0.0009
PTPRZ1	protein tyrosine phosphatase, receptor type Z1 [Source:NCBI gene;Acc:396403]	0.598217	2.22247	1.893423	0.0013
ENSGALG00000042647		1.17216	4.37283	1.899398	0.00085
FBXO15	F-box protein 15 [Source:HGNC Symbol;Acc:HGNC:13617]	1.38226	5.17432	1.90434	0.01205
STMN2	stathmin 2 [Source:NCBI gene;Acc:396095]	7.12966	26.9443	1.918075	0.00025
ENSGALG00000047143	heme binding protein 2 [Source:NCBI gene;Acc:424441]	1.01159	3.84777	1.927398	0.0047
ENSGALG00000052741		3.14908	12.0016	1.930224	0.0017
GFI1B	growth factor independent 1B transcriptional repressor [Source:NCBI gene;Acc:395977]	3.67752	14.0426	1.933005	0.00025
ENSGALG00000000584	protein tyrosine phosphatase, receptor type, V, pseudogene [Source:NCBI gene;Acc:421174]	2.08989	8.05668	1.946758	0.00005
PPP4R4	protein phosphatase 4 regulatory subunit 4 [Source:HGNC Symbol;Acc:HGNC:23788]	1.69401	6.53389	1.9475	0.00395
HSD17B2	hydroxysteroid 17-beta dehydrogenase 2 [Source:HGNC Symbol;Acc:HGNC:5211]	1.04409	4.07781	1.965548	0.00375
VSIG1	V-set and immunoglobulin domain containing 1 [Source:NCBI gene;Acc:414795]	0.843273	3.32819	1.980666	0.01745
MYL1	myosin, light chain 1, alkali; skeletal, fast [Source:NCBI gene;Acc:396470]	7.25568	29.0408	2.000898	0.00005
C1QTNF8	C1q and TNF related 8 [Source:HGNC Symbol;Acc:HGNC:31374]	0.991744	3.9725	2.002008	0.0128
VIT	vitrin [Source:NCBI gene;Acc:421471]	0.380599	1.54184	2.018309	0.00565
KSR2	kinase suppressor of ras 2 [Source:HGNC Symbol;Acc:HGNC:18610]	0.34473	1.40227	2.024225	0.0029
ENSGALG00000048562	leukocyte immunoglobulin-like receptor subfamily A member 2 [Source:NCBI gene;Acc:112531107]	0.499203	2.09935	2.072244	0.01905
MYH1A	myosin, heavy chain 1A, skeletal muscle (similar to human myosin, heavy chain 1, skeletal muscle,	0.337394	1.43705	2.090604	0.00155

	adult) [Source:NCBI gene;Acc:417309]				
DYNLRB2	dynein light chain roadblock-type 2 [Source:NCBI gene;Acc:415802]	3.45617	14.8911	2.107204	0.04175
C12orf74	chromosome 12 open reading frame 74 [Source:HGNC Symbol;Acc:HGNC:27887]	2.89371	12.5624	2.11812	0.00015
C1QL2	complement C1q like 2 [Source:HGNC Symbol;Acc:HGNC:24181]	0.367676	1.61452	2.134598	0.0296
ACTN2	actinin alpha 2 [Source:NCBI gene;Acc:396263]	0.970971	4.32871	2.156437	0.0005
ENSGALG00000009552	galectin-related protein-like [Source:NCBI gene;Acc:423277]	1.32337	6.02213	2.186057	0.02345
TMOD1	tropomodulin 1 [Source:NCBI gene;Acc:395883]	0.585538	2.75502	2.234228	0.0037
CEP57	centrosomal protein 57 [Source:HGNC Symbol;Acc:HGNC:30794]	12.8572	60.597	2.23667	0.00005
KRT5	keratin 5 [Source:NCBI gene;Acc:407779]	1.08347	5.17573	2.256103	0.00035
LIPC	lipase C, hepatic type [Source:HGNC Symbol;Acc:HGNC:6619]	1.59263	7.94806	2.319192	0.01825
ENSGALG00000044935		1.70077	8.59441	2.337211	0.0018
ENSGALG00000003164		1.35464	6.86153	2.340621	0.02775
ENSGALG00000028612	myosin, heavy chain 1G, skeletal muscle (similar to human myosin, heavy chain 1, skeletal muscle, adult) [Source:NCBI gene;Acc:427789]	0.478833	2.47838	2.371803	0.0002
HTR2A	5-hydroxytryptamine receptor 2A [Source:NCBI gene;Acc:428070]	2.52802	13.2924	2.394522	0.00005
C2CD4C	C2 calcium dependent domain containing 4C [Source:HGNC Symbol;Acc:HGNC:29417]	0.55728	3.42315	2.61885	0.0044
EPYC	epiphycan [Source:NCBI gene;Acc:417890]	0.263784	1.6686	2.661209	0.0414
ACAN	aggrecan [Source:NCBI gene;Acc:395798]	0.257834	1.77139	2.780367	0.00035
ENSGALG00000048310		1.10318	7.6423	2.792339	0.004
CAPN9	calpain 9 [Source:HGNC Symbol;Acc:HGNC:1486]	0.240566	1.68827	2.811041	0.00645
INSC	INSC spindle orientation adaptor protein [Source:HGNC Symbol;Acc:HGNC:33116]	0.62546	4.48496	2.842106	0.01445

ACTC1	actin, alpha, cardiac muscle 1 [Source:NCBI gene;Acc:423298]	1.90222	13.7136	2.84985 1	0.0002
ENSGALG00000005 180		0.43327 3	3.15684	2.86513 3	0.0019 5
C3H1orf95	chromosome 3 C1orf95 homolog [Source:NCBI gene;Acc:421315]	0.91541 2	6.80787	2.89471	0.0381 5
ENSGALG00000016 475	long intergenic non-protein coding RNA 954 [Source:NCBI gene;Acc:421956]	0.93295 3	7.03973	2.91564 4	0.0004
MYH1C	myosin, heavy chain 1C, skeletal muscle (similar to human myosin, heavy chain 1, skeletal muscle, adult) [Source:NCBI gene;Acc:417310]	0.57042 5	4.45332	2.96477 2	0.0000 5
CYTL1	cytokine like 1 [Source:HGNC Symbol;Acc:HGNC:24435]	1.08496	8.53151	2.97515 9	0.0018
COL9A1	collagen type IX alpha 1 chain [Source:NCBI gene;Acc:771873]	0.43538 1	3.62858	3.05905 5	0.0003
MYOZ2	myozenin 2 [Source:NCBI gene;Acc:422682]	0.26003 2	2.18273	3.06937 3	0.0240 5
ENSGALG00000046 817		0.56496	6.20822	3.45795 9	0.0114
MYOT	myotilin [Source:HGNC Symbol;Acc:HGNC:12399]	0.12615 6	1.50378	3.57531 3	0.0165 5
MYL3	myosin, light chain 3, alkali; ventricular, skeletal, slow [Source:NCBI gene;Acc:396067]	0.33794 9	4.48486	3.73018 6	0.0034 5
MPZ	myelin protein zero [Source:NCBI gene;Acc:100859605]	0.28512 2	3.96578	3.79795 3	0.0041
COL2A1	collagen type II alpha 1 chain [Source:NCBI gene;Acc:395069]	0.18498 1	3.74789	4.34063	0.0001
MB	myoglobin [Source:NCBI gene;Acc:418056]	0.57929 3	12.585	4.44126 8	0.0088
ENSGALG00000014 184	mothers against decapentaplegic homolog 2-like [Source:NCBI gene;Acc:769000]	20.9813	0	-Inf	0.0000 5
ENSGALG00000030 707	chromosome Z open reading frame, human C18orf25 pseudogene [Source:NCBI gene;Acc:100858742]	1.9264	0	-Inf	0.0175
ENSGALG00000031 327	chromodomain helicase DNA binding protein 1 [Source:NCBI gene;Acc:374195]	6.39203	0	-Inf	0.0000 5
ENSGALG00000034 488	zinc finger RNA-binding protein-like [Source:NCBI gene;Acc:427010]	8.95222	0	-Inf	0.0000 5
ENSGALG00000035 780	E3 ubiquitin-protein ligase KCMF1-like [Source:NCBI gene;Acc:431003]	10.6438	0	-Inf	0.0000 5

ENSGALG00000041221		28.4391	0	-Inf	0.00005
ENSGALG00000046789		2.69881	0	-Inf	0.00005
ENSGALG00000046840		3.72965	0	-Inf	0.01195
ENSGALG00000049219		1.56062	0	-Inf	0.00005
ENSGALG00000050250	histone cluster 1, H4d [Source:NCBI gene;Acc:427884]	4.86031	0	-Inf	0.0123
ENSGALG00000050647	ras GTPase-activating protein 1-like [Source:NCBI gene;Acc:112530485]	10.0998	0	-Inf	0.00005
ENSGALG00000053074		2.14759	0	-Inf	0.00065
RAD9B	RAD9 checkpoint clamp component B [Source:HGNC Symbol;Acc:HGNC:21700]	1.34879	0	-Inf	0.00005
SMAD7B	TGF-beta signal pathway antagonist Smad7 [Source:NCBI gene;Acc:395544]	1.36832	0	-Inf	0.00005
UBAP2	ubiquitin associated protein 2 [Source:NCBI gene;Acc:407091]	11.0051	0	-Inf	0.00005

Table S2. Gene ontology enrichment of resistant H5N1 chicken line versus susceptible H5N1 chicken line in the lung for genes with $p < 0.05$ and Log2-transformation of the normalization of resistant/susceptible ≥2 (1)

Domain	Term.ID	Term name	overlap.size(Q∩T)	adjusted.p-value
MF	GO:0008009	chemokine activity	6	0.000674
MF	GO:0042379	chemokine receptor binding	6	0.00108
MF	GO:0048018	receptor ligand activity	17	0.0116
MF	GO:0001664	G-protein coupled receptor binding	10	0.0199
MF	GO:0030545	receptor regulator activity	17	0.0213
MF	GO:0005125	cytokine activity	10	0.0271
MF	GO:0097367	carbohydrate derivative binding	120	0.0416
BP	GO:0009607	response to biotic stimulus	82	4.15E-23
BP	GO:0051707	response to other organism	76	7.33E-21
BP	GO:0043207	response to external biotic stimulus	76	1.02E-20
BP	GO:0006952	defense response	97	7.78E-19
BP	GO:0002376	immune system process	139	4.51E-17
BP	GO:0009605	response to external stimulus	128	4.41E-16
BP	GO:0006955	immune response	76	9.15E-14
BP	GO:0009617	response to bacterium	46	4.26E-12
BP	GO:0045087	innate immune response	45	1.11E-11
BP	GO:0002682	regulation of immune system process	83	2.05E-11
BP	GO:0098542	defense response to other organism	41	2.24E-11

BP	GO:0002237	response to molecule of bacterial origin	32	1.35E-10
BP	GO:0006954	inflammatory response	47	9.94E-10
BP	GO:0009615	response to virus	33	1.29E-09
BP	GO:0034097	response to cytokine	60	1.71E-09
BP	GO:0032496	response to lipopolysaccharide	29	1.95E-09
BP	GO:0001816	cytokine production	52	1.28E-08
BP	GO:0019221	cytokine-mediated signaling pathway	33	1.95E-08
BP	GO:0002684	positive regulation of immune system process	57	2.91E-08
BP	GO:0031347	regulation of defense response	45	3.94E-08
BP	GO:0050896	response to stimulus	348	1.01E-07
BP	GO:0006950	response to stress	159	1.55E-07
BP	GO:0051704	multi-organism process	95	2.25E-07
BP	GO:0050776	regulation of immune response	43	2.49E-07
BP	GO:0070887	cellular response to chemical stimulus	142	3.38E-07
BP	GO:0051607	defense response to virus	24	4.1E-07
BP	GO:0045088	regulation of innate immune response	25	6.44E-07
BP	GO:0071345	cellular response to cytokine stimulus	52	1.04E-06
BP	GO:0002252	immune effector process	45	1.13E-06
BP	GO:0042221	response to chemical	177	1.39E-06
BP	GO:0001817	regulation of cytokine production	44	1.98E-06
BP	GO:0010033	response to organic substance	82	3.65E-06
BP	GO:0071222	cellular response to lipopolysaccharide	18	2.02E-05
BP	GO:0001819	positive regulation of cytokine production	21	0.000024
BP	GO:0034341	response to interferon-gamma	9	2.45E-05
BP	GO:0071219	cellular response to molecule of bacterial origin	18	3.21E-05
BP	GO:0045089	positive regulation of innate immune response	21	3.92E-05
BP	GO:1990266	neutrophil migration	14	4.52E-05
BP	GO:0050778	positive regulation of immune response	34	4.93E-05
BP	GO:0071216	cellular response to biotic stimulus	19	5.48E-05
BP	GO:0097530	granulocyte migration	16	6.69E-05
BP	GO:0031349	positive regulation of defense response	27	7.84E-05
BP	GO:0050663	cytokine secretion	20	9.03E-05
BP	GO:0002224	toll-like receptor signaling pathway	16	0.000167
BP	GO:0071310	cellular response to organic substance	73	0.000172
BP	GO:0032101	regulation of response to external stimulus	46	0.000173
BP	GO:0071346	cellular response to interferon-gamma	8	0.000228
BP	GO:0080134	regulation of response to stress	68	0.000285
BP	GO:0048584	positive regulation of response to stimulus	89	0.000314
BP	GO:0002831	regulation of response to biotic stimulus	16	0.000338
BP	GO:0002218	activation of innate immune response	17	0.000368

BP	GO:0008219	cell death	93	0.00039
BP	GO:0050707	regulation of cytokine secretion	16	0.000405
BP	GO:0060333	interferon-gamma-mediated signaling pathway	8	0.000545
BP	GO:0071621	granulocyte chemotaxis	14	0.000579
BP	GO:0030593	neutrophil chemotaxis	12	0.00065
BP	GO:0002274	myeloid leukocyte activation	14	0.00139
BP	GO:0002758	innate immune response-activating signal transduction	17	0.00155
BP	GO:0002221	pattern recognition receptor signaling pathway	17	0.00155
BP	GO:0006915	apoptotic process	85	0.00174
BP	GO:0012501	programmed cell death	86	0.00186
BP	GO:0045321	leukocyte activation	44	0.00216
BP	GO:0050708	regulation of protein secretion	25	0.00252
BP	GO:0048518	positive regulation of biological process	217	0.00254
BP	GO:0002791	regulation of peptide secretion	26	0.00261
BP	GO:0032635	interleukin-6 production	14	0.00293
BP	GO:0033993	response to lipid	29	0.0032
BP	GO:0048583	regulation of response to stimulus	145	0.00335
BP	GO:0030595	leukocyte chemotaxis	19	0.00361
BP	GO:0050900	leukocyte migration	24	0.00414
BP	GO:0060759	regulation of response to cytokine stimulus	9	0.00475
BP	GO:0001775	cell activation	49	0.00491
BP	GO:0002253	activation of immune response	24	0.00597
BP	GO:1902622	regulation of neutrophil migration	7	0.00823
BP	GO:0042981	regulation of apoptotic process	72	0.00829
BP	GO:0070098	chemokine-mediated signaling pathway	6	0.0085
BP	GO:0097529	myeloid leukocyte migration	17	0.0088
BP	GO:0043900	regulation of multi-organism process	26	0.0102
BP	GO:0010941	regulation of cell death	76	0.0104
BP	GO:0002232	leukocyte chemotaxis involved in inflammatory response	3	0.0108
BP	GO:0072593	reactive oxygen species metabolic process	8	0.0119
BP	GO:1901700	response to oxygen-containing compound	62	0.0122
BP	GO:0043067	regulation of programmed cell death	72	0.0132
BP	GO:1990868	response to chemokine	6	0.0133
BP	GO:1990869	cellular response to chemokine	6	0.0133
BP	GO:0002460	adaptive immune response based on somatic recombination of immune receptors built from immunoglobulin superfamily domains	15	0.0138
BP	GO:0032735	positive regulation of interleukin-12 production	7	0.0162
BP	GO:0002250	adaptive immune response	17	0.0163
BP	GO:0051716	cellular response to stimulus	281	0.0164

BP	GO:0002443	leukocyte mediated immunity	19	0.0173
BP	GO:0016053	organic acid biosynthetic process	26	0.0206
BP	GO:0046394	carboxylic acid biosynthetic process	26	0.0206
BP	GO:0019724	B cell mediated immunity	10	0.0208
BP	GO:0016064	immunoglobulin mediated immune response	10	0.0208
BP	GO:1903555	regulation of tumor necrosis factor superfamily cytokine production	8	0.021
BP	GO:0006959	humoral immune response	12	0.0253
BP	GO:1903530	regulation of secretion by cell	37	0.0256
BP	GO:0002756	MyD88-independent toll-like receptor signaling pathway	4	0.0262
BP	GO:0071706	tumor necrosis factor superfamily cytokine production	8	0.0269
BP	GO:0048525	negative regulation of viral process	5	0.0273
BP	GO:0019730	antimicrobial humoral response	6	0.0277
BP	GO:2001057	reactive nitrogen species metabolic process	9	0.0296
BP	GO:0051240	positive regulation of multicellular organismal process	73	0.0305
BP	GO:1903557	positive regulation of tumor necrosis factor superfamily cytokine production	6	0.0318
BP	GO:0032103	positive regulation of response to external stimulus	11	0.032
BP	GO:0009306	protein secretion	27	0.0332
BP	GO:0001959	regulation of cytokine-mediated signaling pathway	8	0.0342
BP	GO:0002230	positive regulation of defense response to virus by host	5	0.0348
BP	GO:0031663	lipopolysaccharide-mediated signaling pathway	8	0.0352
BP	GO:0023052	signaling	248	0.0361
BP	GO:0032675	regulation of interleukin-6 production	12	0.0373
BP	GO:0007154	cell communication	250	0.0396
BP	GO:0065007	biological regulation	425	0.043
BP	GO:0032501	multicellular organismal process	250	0.049
CC	GO:0005576	extracellular region	45	2.43E-05
CC	GO:0044421	extracellular region part	37	9.83E-05
CC	GO:0005615	extracellular space	29	0.00334

(1) Log2(FC): For a gene, calculated by 'Log2(Experiment) - Log2(Control)'. '(Experiment)' means a mean FPKM of experimental group, and '(Control)' means a mean FPKM of control group.

MF: molecular function, BP: biological process, and CC: cellular component.

Table S3: All of the DEGs related to the cytokine-cytokine receptor interaction pathways between resistant and susceptible H5N1 induced Ri chicken line of the lung (p-value). This data showed that changes in gene expression for 33 DEGs in H5N1 induced Ri chicken lines.

Gene	Descriptions	Fold change	Accession No
CCL4	C-C motif chemokine ligand 4 [Source:NCBI gene;Acc:395468]	-3.968	NM_204720
CCL19	C-C motif chemokine ligand 19 [Source:NCBI gene;Acc:427406]	-3.776	NM_001302168
IFNG	interferon gamma [Source:NCBI gene;Acc:396054]	-3.337	NM_205149
IL6	interleukin 6 [Source:NCBI gene;Acc:395337]	-3.259	NM_204628
IL8L1	interleukin 8-like 1 [Source:NCBI gene;Acc:395872]	-2.694	NM_205018
CSF2RA	colony stimulating factor 2 receptor alpha subunit [Source:NCBI gene;Acc:418666]	-2.201	NM_001312662
CXCR1	C-X-C motif chemokine receptor 1 [Source:NCBI gene;Acc:430652]	-2.181	NM_001282432
CCL17	C-C motif chemokine ligand 17 [Source:NCBI gene;Acc:415652]	-2.162	NM_001293309
IL8	interleukin 8-like 2 [Source:NCBI gene;Acc:396495]	-2.075	NM_205498
IL1R2	interleukin 1 receptor type 2 [Source:HGNC Symbol;Acc:HGNC:5994]	-1.935	XM_015277810
IL1RL1	interleukin 1 receptor like 1 [Source:NCBI gene;Acc:374136]	-1.902	NM_001024590
IL18	interleukin 18 [Source:NCBI gene;Acc:395312]	-1.822	NM_204608
IL1B	interleukin 1, beta [Source:NCBI gene;Acc:395196]	-1.736	NM_204524
TNFRSF11B	TNF receptor superfamily member 11b [Source:NCBI gene;Acc:378803]	-1.726	NM_001033641
IL13RA2	interleukin 13 receptor subunit alpha 2 [Source:NCBI gene;Acc:422219]	-1.684	NM_001048078
ENSGALG00000025881	colony stimulating factor 2 receptor beta common subunit [Source:NCBI gene;Acc:101750812]	-1.671	XM_015288212
CD40	CD40 molecule [Source:NCBI gene;Acc:395385]	-1.507	NM_204665

Gene	Descriptions	Fold change	Accession No
CRLF2	cytokine receptor like factor 2 [Source:HGNC Symbol;Acc:HGNC:14281]	-1.477	XM_015277738
ENSGALG00000050441	cytokine receptor common subunit beta-like [Source:NCBI gene;Acc:771315]	-1.428	XM_001234608
CSF1	colony stimulating factor 1 [Source:NCBI gene;Acc:100499189]	-1.390	NM_001193295
TNFRSF6B	TNF receptor superfamily member 6b [Source:HGNC Symbol;Acc:HGNC:11921]	-1.386	XM_004947134
CCR2	C-C motif chemokine receptor 2 [Source:NCBI gene;Acc:420696]	-1.359	NM_001045835
IL2RA	interleukin 2 receptor subunit alpha [Source:NCBI gene;Acc:395294]	-1.315	NM_204596
IL18RAP	interleukin 18 receptor accessory protein [Source:HGNC Symbol;Acc:HGNC:5989]	-1.314	XM_015277818
IL10RB	interleukin 10 receptor subunit beta [Source:NCBI gene;Acc:395663]	-1.307	NM_204857
IL20RA	interleukin 20 receptor subunit alpha [Source:HGNC Symbol;Acc:HGNC:6003]	-1.228	XM_015284185
TNFSF13B	tumor necrosis factor superfamily member 13b [Source:NCBI gene;Acc:374229]	-1.222	NM_204327
ACVR2A	activin A receptor type 2A [Source:NCBI gene;Acc:396324]	-1.197	NM_205367
ACKR4	atypical chemokine receptor 4 [Source:HGNC Symbol;Acc:HGNC:1611]	-1.085	XM_025147497
TNFRSF1B	TNF receptor superfamily member 1B [Source:NCBI gene;Acc:395083]	-1.039	NM_204439
IL17RA	interleukin 17 receptor A [Source:NCBI gene;Acc:418158]	-1.028	NM_001318988
EDA2R	ectodysplasin A2 receptor [Source:NCBI gene;Acc:422166]	-1.009	NM_001083360
CXCL14	C-X-C motif chemokine ligand 14 [Source:NCBI gene;Acc:395451]	1.349	NM_204712
